# Just ask Siri? A pilot study comparing smartphone digital assistants and laptop Google searches for smoking cessation advice

**DOI:** 10.1371/journal.pone.0194811

**Published:** 2018-03-28

**Authors:** Matt Boyd, Nick Wilson

**Affiliations:** 1 Adapt Research Ltd, Reefton, New Zealand; 2 Department of Public Health, Univeristy of Otago, Wellington, New Zealand; The Chinese University of Hong Kong, HONG KONG

## Abstract

**Objective:**

To compare voice-activated internet searches by smartphone (two digital assistants) with laptop ones for information and advice related to smoking cessation.

**Design:**

Responses to 80 questions on a range of topics related to smoking cessation (including the FAQ from a NHS website), compared for quality.

**Setting:**

Smartphone and internet searches as performed in New Zealand.

**Main outcome measures:**

Ranked responses to the questions.

**Results:**

Google laptop internet searches came first (or first equal) for best quality smoking cessation advice for 83% (66/80) of the responses. Voiced questions to Google Assistant (“OK Google”) came first/first equal 76% of the time vs Siri (Apple) at 28%. Google and Google Assistant were statistically significantly better than Siri searches (odds ratio 12.4 and 8.5 respectively, p<0.0001 in each comparison). When asked FAQs from the National Health Service website, or to find information the Centers for Disease Control has made videos on, the best search results used expert sources 59% (31/52) of the time, “some expertise” (eg, Wikipedia) 18% of the time, but also magazines and other low quality sources 19% of the time. Using all three methods failed to find relevant information 8% (6/80) of the time, with Siri having the most failed responses (53% of the time).

**Conclusion:**

Google internet searches and Google Assistant were found to be significantly superior to the Siri digital assistant for smoking cessation information. While expert content was returned over half the time, there is still substantial room for improvement in how these software systems deliver smoking cessation advice.

## Introduction

The internet is widely used for obtaining health-related information and advice. For example, in the United Kingdom, 41% of internet users report going online to find information for health-related issues, with about half of these (22% of all users) having done so in the previous week [1]. But many people are also wary of the information they find online and value trusted sources [2]. Improving search engine functionality offers a potential solution. For example, Google is cooperating with Mayo Clinic physicians to curate and check health data that is added to the database it uses for instant search results [3]. Similarly, National Health Service (NHS) England is working with Microsoft and Google to increase the visibility of NHS content online [4].

With increasing smartphone use there is also a particular case for studying health information obtainable with digital assistants on smartphones. Present literature on digital assistant use is very limited [5–7]. and there appears to be no published research on the use of these tools in providing information or advice on smoking cessation. Therefore we aimed to assess the current situation using the digital assistants Siri and Google Assistant (GA) and to compare these with internet searches.

## Methods

### Selection of digital assistants

Siri (Apple) and GA (Google) were selected because they were in common use as personal digital assistants at the time of the Pilot study in October 2017 [5, 6].

### Selection of questions

The first set of questions (n = 35) were adapted from the most detailed “frequently asked questions (FAQ)” we could identify: that of the UK National Health Service (NHS) smokefree website [8]. The specific questions are listed in S1 Appendix, including slight modifications so they are relevant to an international audience.

The next set of questions (n = 17) were related to the most comprehensive list of short videos on smoking-related disease that we could identify: those produced by the Centers for Disease Control and Prevention (CDC) in the USA for the “Tips From Former Smokers” Campaign [9].

The final set of questions (n = 28) were those devised by us to test responses to a range of features such as, finding smoking-related pictures, diagrams, instructional videos; and navigating to the nearest service/retailer for quitting-related products.

### Data collection

Data were collected independently by both researchers on a pre-designed form and each independently conducted their own quality grading and rankings (internet search vs GA vs Siri).

For speaking into the smartphones, a maximum of three attempts were made per question by the two authors (both of whom had New Zealand accents). The smartphones used were an iPhone 5S and an iPhone 7, with settings for “English (New Zealand)”. For Google searches on laptops, the site used was that for New Zealand (https://www.Google.co.nz/) and using Google Chrome. Only the first non-advertisement link or information returned was considered in the analysis. All searches were conducted in October 2017 with both researchers being located in New Zealand (in the capital city and a small rural town, 250 km apart).

### Hierarchy of information/advice quality

In independently grading the quality of the information and advice, we used the following hierarchy:

Grade A: Health agencies which had medical expertise whether local or international (eg, Ministry of Health, the national Quitline service, the NHS, CDC, universities, and hospitals).

Grade B: Sites with “some expertise”. Examples were Wikipedia and commercially orientated medical sites such as WebMD, or certified clinicians giving information directly.

Grade C: Online news items, online magazines and internet sites run by individuals and non-health organisations.

### Analysis

Inter-rater agreement was calculated on the ratings of quality of the content and which tools were best or equal best in answering each question. The frequency with which the three search tools provided the best information was compared using odd ratios.

## Results

The tools frequently returned different search results to the two raters. On the 55 occasions that the best quality result was the same for both raters, there was 100% concordance of the raters’ grading of quality of the information (grades: A, B or C).

Cohen’s kappa was calculated for the level of observer agreement for ranking which tool had returned the best or best equal information. There were eight possible ranking choices for each question (one tool being best alone, or combinations of best equal, or none) and kappa was 0.45 –when blinded, showing moderate agreement. This was surely lowered by instances where the search results returned were different between raters. When instances where the content returned by the best rated tool was the same, kappa rose to 0.56.

A laptop-based Google search provided the best or equal best information 83% (66/80) of the time (Table 1, see also S1 Appendix for specific results). GA was the better digital assistant, with 76% of the best (or best equal) responses, compared to Siri (28%). All three search approaches were classified as equally successful for only 18 questions (22%). The results for Google searches were not statistically significantly better than GA, but were considerably better than Siri, odds ratio (OR) = 12.4 (95% CI = 5.8–26.5, p<0.0001). GA was better than Siri with OR = 8.5 (4.2–17.3, p<0.0001).

**Table 1:** Results for smoking cessation information and advice provided by Siri, Google Assistant and Google searches (see S1 Appendix for question specific results).

Topic (for all n = 80 questions unless stated otherwise)	Typed Google search on a laptop	Google Assistant (GA)	Siri
Provided the best advice (first or first equal)	83% (66^#^/80)	76% (61/80)	28% (22/80) **
Provided the best advice (first or first equal) for the NHS FAQ questions (n = 35 questions)	90% (32/35)	79% (28/35)	49% (17/35) **
Provided the best advice (first or first equal) for the questions around accessing videos (n = 17 questions)	79% (14/17)	85% (15/17)	0% (0/17) **
Provided the best information (first or first equal) for the pictures, locations and other functionality questions (n = 28 questions)	75% (21/28)	66% (19/28)	18% (5/28) **
Failed to provide any useful information	9% (7/80)	14% (12/80)	53% (42/80) **
First response was one or more advertisements	21% (17/80)	28% (22/80)	8% (6/80) *
Mean number of advertisements prior to a non-advertising response	0.4 adverts	0.6 adverts	0.3 adverts
Answer was from an expert source (grade A) (n = 52 questions from the NHS/CDC)	52% (27/52)	49% (26/52)	24% (13/52) *
Answer was from a semi- expert source (grade B) (n = 52 questions from the NHS/CDC)	22% (12/52)	20% (11/52)	13% (7/52)
Answer was from a non-expert source (grade C) (n = 52 questions from the NHS/CDC)	22% (12/52)	24% (13/52)	13% (7/52)

Notes

^#^ mean of two raters rounded up to next whole number; statistical tests compared GA to Siri:

*p<0.01

**p<0.001

Google searches also had the lowest outright failure rate of providing no useful response for 9% (7/80) of the questions, compared to GA (14%, 12/80) and Siri (53%, 42/80) with no significant differences between the former and GA, however Google was superior to Siri (p<0.0001), as was GA (p<0.0001). All three devices failed on only 8% (6/80) questions.

For assessing response quality, we considered just the questions relating to the NHS 35 FAQs and also those relating to the CDC’s set of 17 videos on smoking cessation. Taking just the best result for each of these 52 questions, 59% (31/52) of the search questions were answered with a best answer that we determined to be expert sources. These included the CDC (n = 10), Cancer.org (n = 6), NHS (n = 4), and a range of other medical expert-endorsed sites eg, hospitals, specialist clinics, and universities. Around a fifth (18%, 10/52) of searches provided websites with “some expertise” such as Wikipedia articles and commercially orientated ones (eg, private medical clinics), and 19% of searches provided only news items or magazine articles.

## Discussion

### Main findings and interpretation

Our search results were encouraging in terms of the usefulness of the information provided, with nearly 60% of searches returning expert content on at least one tool, and Google and GA returning expert content about half the time. However, all search modalities could improve on the chances of finding expert information.

Our results are consistent however, with the only other reported health-related study, which was undertaken in 2015/2016 [7]. It found that Siri and other smartphone assistants sometimes trivialised important general health inquiries or failed to provide appropriate information. We found that all tools had trouble finding gay and lesbian-specific information, Siri was poor when videos were requested by content, and all three tools sometimes returned magazine or blog content instead of professional health advice.

The responses sometimes included a useful Google summary box, and/or a diagram. The summary was often read out verbally by the digital assistants and this has obvious advantages for people with disabilities or some situations such as when the questioner is doing other activities. There was notable variation in the search results between the two researchers. For example, when asked to find an antismoking advertisement, rater A was directed to a New Zealand public health campaign advertisement, while rater B was shown a Youtube video of the ‘top 40 scariest antismoking ads’ from around the world (S1 Appendix). This variation may reflect the impact of location, Google search history, demographics, ongoing changes in website traffic and website links on search algorithms.

### Study strengths and limitations

A strength is that this study is the first to consider smartphone digital assistants for the provision of smoking cessation information and advice. It also used questions derived from expert sources (NHS and CDC) and tested a wide range of smartphone functionalities with the two researchers collecting data independently. But a possible limitation is that our results might be superior to questions asked in the real world since we used reasonably precise wording and terms, as opposed to slang words or colloquialisms that some of the public might use. On the other hand, we only considered the first result returned in each search list, and there were often superior sites listed after the initial sites.

### Potential research implications

These pilot results demonstrate that a range of useful information is returned to users of digital assistants when asking for smoking cessation advice. This suggests that a larger study of actual smokers wanting to quit is warranted. The larger study could investigate the user experience as well as the quality of the information returned by digital assistants. In the meantime, however, software designers and health authorities should continue to work together to improve search functionality, as is starting to happen in some localities [3, 4].

## Conclusions

Google internet searches and Google Assistant were found in this pilot study to be significantly superior to the Siri digital assistant for sourcing smoking cessation content. While expert content was returned over half the time, there is still substantial room for improvement in how these software systems deliver smoking cessation advice.

## Supporting information

S1 Appendix Search results by question. (DOCX)
