# Supplementary material for: Just ask Siri? A pilot study comparing smartphone digital assistants and laptop Google searches for smoking cessation advice
Source: PLoS One. 2018 Mar 28;13(3):e0194811. doi: 10.1371/journal.pone.0194811 (PMC5874038; doi:10.1371/journal.pone.0194811)
Supplement: S1 Appendix — (DOCX) [file pone.0194811.s001.docx]

**Appendix 1: Table A - Search results by question**

| **Question** | **Typed Google search on a laptop** | | | **Google Assistant’s (GA) response with all questions preceded by “Okay Google”** | | | | **Siri’s response (with all questions preceded by “Hey Siri”)** | | | | **Rater 1 ranking** | **Rater 2 ranking** |
| --- | --- | --- | --- | --- | --- | --- | --- | --- | --- | --- | --- | --- | --- |
|  | **Ads** | **Quality** | **Details** | **spoken sentences** | **Ads** | **Quality** | **Details** | **spoken sentences** | **Ads** | **Quality** | **Details** |  |  |
| Q1:  Is it too late to stop smoking | 1 | C | TIME magazine health section (blog). Gives information on outcomes for those over 60 who quit: http://healthland.time.com/2012/06/13/its-never-too-late-to-quit-smoking-study-finds/ | 0 | 4 | C | Top link is to: TIME magazine health section (blog). Gives information on outcomes for those over 60 who quit: http://healthland.time.com/2012/06/13/its-never-too-late-to-quit-smoking-study-finds/ | 1 | 0 | Fail | Says: “interesting question Matt” (tried 3 times) | Google = GA > Siri |  |
| Q1 | 0 | C | TIME magazine health section (blog). Gives information on outcomes for those over 60 who quit: http://healthland.time.com/2012/06/13/its-never-too-late-to-quit-smoking-study-finds/ | 0 | 4 | C | Top link is to: TIME magazine health section (blog). Gives information on outcomes for those over 60 who quit: http://healthland.time.com/2012/06/13/its-never-too-late-to-quit-smoking-study-finds/ | 0 | 0 | Fail | Says: “interesting question Nick” (tried 3 times) |  | Google = GA > Siri (fail) |
| Q2:  Will I gain weight when I stop smoking | 0 | A | Google information box appears first, saying people gain 10 pounds (cites American Cancer Society expert, grade A). Then the link in the box is to WebMD (grade B): http://www.webmd.com/smoking-cessation/features/stopping-weight-gain-while-quitting-smoking#1 | 4 | 0 | A | Speaks 4 sentences which are read from the Google information box. Attributed to American Cancer Society (A). Links to WebMD ‘quitting smoking without weight gain’ | 2 | NA | Fail | Says “you’ll need an app for me to help you with that. You could try searching the app store” (also presents the same text) | Google = GA > Siri (fail) |  |
| Q2 | 0 | A | Google information box appears first, saying people gain 10 pounds (cites American Cancer Society expert, grade A). Then the link in the box is to WebMD (grade B): http://www.webmd.com/smoking-cessation/features/stopping-weight-gain-while-quitting-smoking#1 | 4 | 1 | A | Speaks 4 sentences which are read from the Google information box. Attributed to American Cancer Society (A). Links to WebMD ‘quitting smoking without weight gain’ | 0 | 0 | Fail | Says “you’ll need an app for me to help you with that. You could try searching the app store” (also presents the same text) |  | Google = GA > Siri (fail) |
| Q3:  What are the first steps to quitting smoking | 4 | A | Google information box gives 8 step plan and links to Addictions and recovery.org article on how to quit smoking with academic citations. https://www.addictionsandrecovery.org/quit-smoking-plan.htm | 4 | 3 | A | Says: ‘here is a summary from addictionsandrecovery” then reads 4 of the 8 steps, but out of sequence. Link is to addictionsandrecovery.org article on ‘how to quit smoking’ which is comprehensive, step by step and provides academic citations. | 1 | 4 | A | Says, “here’s what I found on the web for what are the first steps to quitting smoking…” gives a link to: addictionsandrecovery.org after x4 ads. There is also a Google information box listing 8 steps. (same article as Google) | Google = Siri > GA |  |
| Q3 | 3 | A | Google information box gives 8 step plan and links to Addictions and recovery.org article on how to quit smoking with academic citations. https://www.addictionsandrecovery.org/quit-smoking-plan.htm | 4 | 3 | A | Google information box gives 8 step plan and links to Addictions and recovery.org article on how to quit smoking with academic citations. https://www.addictionsandrecovery.org/quit-smoking-plan.htm | 1 | 0 | A | Says, “here’s what I found on the web for what are the first steps to quitting smoking…” gives a link to: addictionsandrecovery.org. There is also a Google information box listing 8 steps. (same article as Google) |  | Google = GA = Siri |
| Q4:  What support for quitting smoking is available to me | 4 | A | First link is to ‘stop smoking’ page at NZ Ministry of Health: http://www.health.govt.nz/your-health/healthy-living/addictions/smoking/stop-smoking | 0 | 3 | A | First link is to ‘stop smoking’ page at NZ Ministry of Health: http://www.health.govt.nz/your-health/healthy-living/addictions/smoking/stop-smoking | 1 | 4 | A | “Ok, I found this on the web for what support…” First link is to NZ Ministry of Health, as per Google. | Google = GA = Siri |  |
| Q4 | 4 | A | First link is to ‘stop smoking’ page at NZ Ministry of Health: http://www.health.govt.nz/your-health/healthy-living/addictions/smoking/stop-smoking | 0 | 4 | A | First link is to ‘stop smoking’ page at NZ Ministry of Health: http://www.health.govt.nz/your-health/healthy-living/addictions/smoking/stop-smoking | 1 | 0 | A | National Cancer Institute in the USA: https://www.cancer.gov/about-cancer/causes-prevention/risk/tobacco/help-quitting-fact-sheet |  | Google = GA > Siri |
| Q5:  What withdrawal symptoms can I expect when I quit smoking | 0 | C | First link is to quit smoking support.com: http://www.quitsmokingsupport.com/withdrawal1.htm it details the symptoms. The site states that ‘it does not make any medical claims’ | 0 | 0 | C | First link is to quit smoking support.com: http://www.quitsmokingsupport.com/withdrawal1.htm it details the symptoms. The site states that ‘it does not make any medical claims’ | 1 | 4 | C | Says: “Ok I found this on the web for what withdrawal…”  First link is to quit smoking support.com: http://www.quitsmokingsupport.com/withdrawal1.htm it details the symptoms. The site states that ‘it does not make any medical claims’ | Google = GA = Siri |  |
| Q5 | 0 | C | First link is to quit smoking support.com: http://www.quitsmokingsupport.com/withdrawal1.htm it details the symptoms. The site states that ‘it does not make any medical claims’ | 0 | 0 | C | First link is to quit smoking support.com: http://www.quitsmokingsupport.com/withdrawal1.htm it details the symptoms. The site states that ‘it does not make any medical claims’ | 1 | 0 | C | Says: “Ok I found this on the web for what withdrawal…”  First link is to quit smoking support.com: http://www.quitsmokingsupport.com/withdrawal1.htm it details the symptoms. The site states that ‘it does not make any medical claims’ |  | Google = GA = Siri |
| Q6:  What recovery symptoms can I expect when I quit smoking | 0 | C | Links to: http://whyquit.com/whyquit/A_Symptoms.html article on nicotine withdrawal and recovery symptoms. Lots of information but Many disclaimers. | 0 | 0 | C | Links to: http://whyquit.com/whyquit/A_Symptoms.html article on nicotine withdrawal and recovery symptoms. Lots of information but Many disclaimers. | 1 | 0 | C | “Here’s what I found on the web for…” Links to: http://whyquit.com/whyquit/A_Symptoms.html article on nicotine withdrawal and recovery symptoms. Lots of information but Many disclaimers. | Google = GA = Siri |  |
| Q6 | 0 | C | Links to: http://whyquit.com/whyquit/A_Symptoms.html article on nicotine withdrawal and recovery symptoms. Lots of information but Many disclaimers. | 0 | 0 | C | Links to: http://whyquit.com/whyquit/A_Symptoms.html article on nicotine withdrawal and recovery symptoms. Lots of information but Many disclaimers. | 1 | 0 | C | “Here’s what I found on the web for…” Links to: http://whyquit.com/whyquit/A_Symptoms.html article on nicotine withdrawal and recovery symptoms. Lots of information but Many disclaimers. |  | Google = GA = Siri |
| Q7:  How can I manage my withdrawal symptoms when I quit smoking | 0 | B | Healthline.com on ‘coping with nicotine withdrawal’: https://www.healthline.com/health/quit-smoking-nicotine-withdrawal article is ‘medically reviewed’ by Dr with PhD. | 0 | 2 | A | First link is to ‘managing withdrawal’ at smokefree.gov.  Which provides thorough advice. | 1 | 0 | A | Says “Here’s what I found on the web for…” also presented as text. Presents a Google information box with 3 options including a Quit line number. First Link is to smokefree.gov ‘understanding withdrawal’ includes a section on ‘you can prepare for withdrawal’ | Siri > GA > Google |  |
| Q7 | 0 | B | Healthline.com on ‘coping with nicotine withdrawal’: https://www.healthline.com/health/quit-smoking-nicotine-withdrawal article is ‘medically reviewed’ by Dr with PhD. | 0 | 1 | B | Healthline.com on ‘coping with nicotine withdrawal’: https://www.healthline.com/health/quit-smoking-nicotine-withdrawal article is ‘medically reviewed’ by Dr with PhD. | 1 | 0 | B | Healthline.com on ‘coping with nicotine withdrawal’: https://www.healthline.com/health/quit-smoking-nicotine-withdrawal article is ‘medically reviewed’ by Dr with PhD. |  | Google = GA = Siri |
| Q8:  How can I manage my recovery symptoms when I quit smoking | 1 | A | First link is to NHS Smokefree ‘what will quitting be like’: https://www.nhs.uk/smokefree/why-quit/what-will-quitting-be-like discusses withdrawal and revovery symptoms | 0 | 1 | C | First link is to: Quitnet.squarespace.com article on ‘the emotional element in quitting smoking’. Gives explanation and options. | 1 | 0 | C | Says “here’s what I found on the web…” First link is to: Quitnet.squarespace.com article on ‘the emotional element in quitting smoking’. Gives explanation and options. | Google > GA = Siri |  |
| Q8 | 0 | A | Information from the addictionsandrecovery.org site | 0 | 1 | A | First link is to NHS Smokefree ‘what will quitting be like’: https://www.nhs.uk/smokefree/why-quit/what-will-quitting-be-like discusses withdrawal and revovery symptoms | 1 | 0 | A | First link is to NHS Smokefree ‘what will quitting be like’: https://www.nhs.uk/smokefree/why-quit/what-will-quitting-be-like discusses withdrawal and revovery symptoms |  | GA = Siri > Google |
| Q9:  How can I manage my cravings when I quit smoking | 0 | A | Google information box lists 4 useful things to try. And links to: smokefree.gov ‘how to manage cravings’: https://smokefree.gov/challenges-when-quitting/cravings-triggers/how-manage-cravings | 5 | 0 | A | Speaks: “here is a summary from smokefree.gov” then lists four steps you can try. Link provided to smokefree.gov article on cravings. | 1 | 0 | A | Says: “here’s what I found on the web…”  Presents a Google information box with four ideas. Link is to smokefree.gov ‘how to manage cravings’ | Google = GA = Siri |  |
| Q9 | 0 | A | Google information box lists 4 useful things to try. And links to: smokefree.gov ‘how to manage cravings’: https://smokefree.gov/challenges-when-quitting/cravings-triggers/how-manage-cravings | 0 | 2 | A | Speaks: “here is a summary from smokefree.gov” then lists four steps you can try. Link provided to smokefree.gov article on cravings. | 2 | 0 | A | Says: “here’s what I found on the web…”  Presents a Google information box with four ideas. Link is to smokefree.gov ‘how to manage cravings’ |  | Google = GA = Siri |
| Q10:  What if I start smoking again after quitting smoking | 0 | C | First link is to ‘how do I recover from a smoking relapse’ https://www.verywell.com/how-do-i-recover-from-a-smoking-relapse-2825216 | 4 | 0 | A | Speaks “here is some information for what happens if you smoke after quitting” link provided to smokefree.gov topic ‘what if you have a cigarette?’ | 1 | 0 | C | Says ‘here’s what I found on the web…’ First link is to ‘how do I recover from a smoking relapse’ https://www.verywell.com/how-do-i-recover-from-a-smoking-relapse-2825216 | GA > Google = Siri |  |
| Q10 | 0 | C | First link is to ‘how do I recover from a smoking relapse’ https://www.verywell.com/how-do-i-recover-from-a-smoking-relapse-2825216 | 0 | 0 | C | First link is to ‘how do I recover from a smoking relapse’ https://www.verywell.com/how-do-i-recover-from-a-smoking-relapse-2825216 | 2 | 0 | C | Says ‘here’s what I found on the web…’ First link is to ‘how do I recover from a smoking relapse’ https://www.verywell.com/how-do-i-recover-from-a-smoking-relapse-2825216 |  | Google = GA = Siri |
| Q11:  I've tried to stop smoking many times, but always fail so what can I do | 0 | B | Healthline news, ‘quitting smoking, expect a lot of failure before you succeed’ article: https://www.healthline.com/health-news/quitting-smoking-expect-failure-before-you-succeed it quotes research studies e.g. BMJ Open and CDC. | 0 | 1 | B | First link is to healthline.com article on ‘quitting smoking, expect a lot of failure before you succeed’ article: https://www.healthline.com/health-news/quitting-smoking-expect-failure-before-you-succeed it quotes research studies e.g. BMJ Open and CDC. | 0 | NA | Fail | Displays “sorry, you can also type what you’re looking for” | Google = GA > Siri (fail) |  |
| Q11 | 0 | B | Healthline news, ‘quitting smoking, expect a lot of failure before you succeed’ article: https://www.healthline.com/health-news/quitting-smoking-expect-failure-before-you-succeed it quotes research studies e.g. BMJ Open and CDC. | 0 | 0 | B | Healthline news, ‘quitting smoking, expect a lot of failure before you succeed’ article: https://www.healthline.com/health-news/quitting-smoking-expect-failure-before-you-succeed it quotes research studies e.g. BMJ Open and CDC. | 2 | 0 | C | Goes to “Recovering from a failed quit smoking attempt and relapse”: http://www.achoice2live.com/recovering-from-a-failed-quit-smoking-attempt-and-relapse/ (it seems to have some reasonable advice) |  | Google = GA > Siri |
| Q12:  I smoke because it helps me deal with stress... so what can I do about this | 0 | B | Link is to Healthline, article on ‘stress, smoking and heart disease’: https://www.healthline.com/health/heart-disease/stress-smoking discusses how smoking causes stress, and ways to cope. Article ‘medically reviewed’ by RN with PhD. | 0 | 0 | A | Links to smokefree.gov article on stress and smoking. Provides alternative ways to deal with stress. Also explains what stress is. | 1 | NA | Fail | Says:”I don’t know how to respond to that” | GA > Google > Siri (fail) |  |
| Q12 | 0 | B | Link is to Healthline, article on ‘stress, smoking and heart disease’: https://www.healthline.com/health/heart-disease/stress-smoking discusses how smoking causes stress, and ways to cope. Article ‘medically reviewed’ by RN with PhD. | 0 | 0 | Fail | Poor advice on a blog post: https://www.xojane.com/healthy/im-not-supposed-be-revealing-heres-how-i-finally-quit-smoking | 0 | 0 | Fail | Says:”I don’t know how to respond to that” |  | Google > GA=Fail, Siri (fail) |
| Q13:  How can I help someone close to me give up smoking | 3 | B | Google information box is first non-ad. It is on understanding nicotine addiction and Tells the user to research, take notes, talk to a health professional, talk to smokers who have quit. Link then goes to WikiHow: http://www.wikihow.com/Persuade-Someone-to-Quit-Smoking topic ‘how to persuade someone to quit smoking’ | 0 | 2 | B | Google information box is first non-ad. It is on understanding nicotine addiction and Tells the user to research, take notes, talk to a health professional, talk to smokers who have quit. Link then goes to WikiHow: http://www.wikihow.com/Persuade-Someone-to-Quit-Smoking topic ‘how to persuade someone to quit smoking’ | 1 | 2 | B | Says ‘OK, I found this on the web for…”  Google information box is first non-ad. It is on understanding nicotine addiction and Tells the user to research, take notes, talk to a health professional, talk to smokers who have quit. Link then goes to WikiHow: http://www.wikihow.com/Persuade-Someone-to-Quit-Smoking topic ‘how to persuade someone to quit smoking’ | Google = GA = Siri |  |
| Q13 | 2 | C | Google information box is first non-ad. It is on understanding nicotine addiction and Tells the user to research, take notes, talk to a health professional, talk to smokers who have quit. Link then goes to WikiHow: http://www.wikihow.com/Persuade-Someone-to-Quit-Smoking topic ‘how to persuade someone to quit smoking’ (no medical input to this site) | 0 | 4 | C | Google information box is first non-ad. It is on understanding nicotine addiction and Tells the user to research, take notes, talk to a health professional, talk to smokers who have quit. Link then goes to WikiHow: http://www.wikihow.com/Persuade-Someone-to-Quit-Smoking topic ‘how to persuade someone to quit smoking’ | 2 | 0 | A | Shows a Google information box and a link to the American Cancer Society on “Helping a Smoker Quit: Do’s and Don’ts”: https://www.cancer.org/healthy/stay-away-from-tobacco/helping-a-smoker-quit.html |  | Siri > Google = GA |
| Q14:  What does smoking do to my body | 0 | A | Google information box gives details on lung damage and COPD and links to the NHS: https://www.nhs.uk/smokefree/why-quit/smoking-health-problems ‘how smoking affects your body’ | 4 | 0 | A | Speaks: “here is a summary from NHS.uk…” gives stats on lung cancer and COPD etc. Provides link to NHS: https://www.nhs.uk/smokefree/why-quit/smoking-health-problems ‘how smoking affects your body’ | 1 | 0 | A | Says: “here’s what I found on the web for what does…” Google information box gives details on lung damage and COPD and links to the NHS: https://www.nhs.uk/smokefree/why-quit/smoking-health-problems ‘how smoking affects your body’ | Google = GA = Siri |  |
| Q14 | 0 | A | Google information box gives details on lung damage and COPD and links to the NHS: https://www.nhs.uk/smokefree/why-quit/smoking-health-problems ‘how smoking affects your body’ | 3 | 1 | A | Speaks: “here is a summary from NHS.uk…” gives stats on lung cancer and COPD etc. Provides link to NHS: https://www.nhs.uk/smokefree/why-quit/smoking-health-problems ‘how smoking affects your body’ | 2 | 0 | B | Links to a medically reviewed online article: https://www.healthline.com/health/smoking/effects-on-body |  | Google = GA > Siri |
| Q15:  What's in a cigarette. | 0 | A | Google information box mentions 4000 ingredients, carcinogens, toxins, and provides a diagram (from Cancer Research UK, A). Then links to http://www.quitsmokingsupport.com/whatsinit.htm article on cigarette ingredients by a Prof of pharmacology (A). | 3 | 0 | A | Speaks “according to quit smoking.com…” and explains how there are 4000 chemicals including arsenic and DDT etc. Provides link to quitsmokingsupport.com also provides a diagram from cancerressearch.uk. | 1 | 0 | B | Says “cigarette is made up of tobacco, filter and rolling paper” – displays same text and provides link to Wikipedia entry on ‘cigarette’. | Google = GA > Siri |  |
| Q15 | 0 | A | Google information box mentions 4000 ingredients, carcinogens, toxins, and provides a diagram (from Cancer Research UK, A). Then links to http://www.quitsmokingsupport.com/whatsinit.htm article on cigarette ingredients by a Prof of pharmacology (A). | 3 | 0 |  | Google information box mentions 4000 ingredients, carcinogens, toxins, and provides a diagram (from Cancer Research UK, A). Then links to http://www.quitsmokingsupport.com/whatsinit.htm article on cigarette ingredients by a Prof of pharmacology (A). | 1 | 0 | B | Says “cigarette is made up of tobacco, filter and rolling paper” – displays same text and provides link to Wikipedia entry on ‘cigarette’. |  | Google = GA > Siri |
| Q16:  Are 'light', 'mild' or 'low tar' cigarettes better for me | 0 | A | Link is to: https://www.cancer.gov/about-cancer/causes-prevention/risk/tobacco/light-cigarettes-fact-sheet which states that No, there is no such thing as a safe cigarette. | 0 | 0 | A | Had to repeat myself once (light = like). But then given link to cancer.gov (NIH) article on light cigarettes and cancer risk. | 1 | 0 | C | Says ‘here’s what I found on the web for…” and gives link to Men’s health article which states, no they are not. | Google > GA > Siri |  |
| Q16 | 0 | A | Link is to: https://www.cancer.gov/about-cancer/causes-prevention/risk/tobacco/light-cigarettes-fact-sheet which states that No, there is no such thing as a safe cigarette. | 0 | 0 | A | Link is to: https://www.cancer.gov/about-cancer/causes-prevention/risk/tobacco/light-cigarettes-fact-sheet which states that No, there is no such thing as a safe cigarette. | 1 | 0 | Fail | Says “I’m not sure I understand” |  | Google = GA > Siri |
| Q17:  Are roll-ups better for me than ordinary cigarettes | 0 | C | Link is to abc australia science page: http://www.abc.net.au/science/articles/2014/07/22/4050221.htm article is by ‘Dr Karl’ and staes that rollies are more harmful. | 0 | 0 | C | Link is to abc australia science page: http://www.abc.net.au/science/articles/2014/07/22/4050221.htm article is by ‘Dr Karl’ and staes that rollies are more harmful. | 1 | 0 | C | Says ‘ok I found this on the web for…’  Link is to abc australia science page: http://www.abc.net.au/science/articles/2014/07/22/4050221.htm article is by ‘Dr Karl’ and staes that rollies are more harmful. | Google = GA = Siri |  |
| Q17 | 0 | B | Link is to an informative site: https://www.verywell.com/the-health-hazards-of-roll-your-own-cigarettes-2825284 (reviewed by a board-certified physician) | 0 | 0 | B | Link is to an informative site: https://www.verywell.com/the-health-hazards-of-roll-your-own-cigarettes-2825284 (reviewed by a board-certified physician) | 2 | 0 | B | Link is to an informative site: https://www.verywell.com/the-health-hazards-of-roll-your-own-cigarettes-2825284 (reviewed by a board-certified physician) |  | Google = GA = Siri |
| Q18:  What is second-hand smoke | 0 | A | Google information box, that explains clearly what it is. Links to American Cancer Society site: https://www.cancer.org/cancer/cancer-causes/tobacco-and-cancer/secondhand-smoke.html article on second hand smoke. | 4 | 0 | A | Speaks “according to american cancer society…” and explains correctly. Provides link to cancer.org article on secondhand smoke. | 1 | 0 | B | Says ‘OK check it out’ and provides information about ‘passive smoking’ from Wikipedia. | Google = GA > Siri |  |
| Q18 | 0 | A | Google information box, that explains clearly what it is. Links to American Cancer Society site: https://www.cancer.org/cancer/cancer-causes/tobacco-and-cancer/secondhand-smoke.html article on second hand smoke | 3 | 0 | A | Speaks “according to american cancer society…” and explains correctly.  Google information box, that explains clearly what it is. Links to American Cancer Society site: https://www.cancer.org/cancer/cancer-causes/tobacco-and-cancer/secondhand-smoke.html article on second hand smoke | 3 | 0 | B | Says ‘OK check it out’ and provides information about ‘passive smoking’ from Wikipedia. |  | Google = GA > Siri |
| Q19:  What are the effects of second-hand smoke | 0 | A | Google information box explains the health risks of SHS, and provides a diagram. Link is to CDC on health effects of SHS: https://www.cdc.gov/tobacco/data_statistics/fact_sheets/secondhand_smoke/health_effects/index.htm | 2 | 0 | A | Speaks “here’s a summary from CDC…” and explains about effects on infants, SIDS, etc. Provides a diagram. Links to CDC fact sheet on secondhand smoke. | 1 | 0 | A | Says ‘OK I found this on the web…’  Provides the same diagram and info as GA but does not speak it aloud. Links to CDC fact sheet on secondhand smoke. | GA = Google = Siri |  |
| Q19 | 0 | A | Google information box explains the health risks of SHS, and provides a diagram. Link is to CDC on health effects of SHS: https://www.cdc.gov/tobacco/data_statistics/fact_sheets/secondhand_smoke/health_effects/index.htm | 3 | 0 | A | Speaks “here’s a summary from CDC…” and explains about effects on infants, SIDS, etc. Provides a diagram. Links to CDC fact sheet on secondhand smoke. | 2 | 0 | A | Says ‘OK I found this on the web…’  Provides the same diagram and info as GA but does not speak it aloud. Links to CDC fact sheet on secondhand smoke. |  | Google = GA = Siri |
| Q20: Is second-hand smoke just as dangerous as smoking a cigarette | 0 | B | Google information box explains that SHS raises risk of lung cancer by 30% and links to WebMD on secondhand smoke risks: http://www.webmd.com/smoking-cessation/effects-of-secondhand-smoke | 3 | 0 | B | Speaks “according to WebMD secondhand smoke has more than 4000 chemicals… it can lead to emphysema and it is bad for your heart” Provides a link to WebMD article on SHS. | 1 | 0 | B | Says ‘here’s what I found on the web for…’ a Google information box is first explains that SHS raises risk of lung cancer by 30% and links to WebMD on secondhand smoke risks: http://www.webmd.com/smoking-cessation/effects-of-secondhand-smoke | GA = Google = Siri |  |
| Q20 | 0 | A | Goes to an informative CDC site on “Secondhand smoke (SHS) facts: https://www.cdc.gov/tobacco/data_statistics/fact_sheets/secondhand_smoke/general_facts/index.htm | 0 | 0 | A | Goes to an informative CDC site on “Secondhand smoke (SHS) facts: https://www.cdc.gov/tobacco/data_statistics/fact_sheets/secondhand_smoke/general_facts/index.htm | 2 | 0 | A | Links to the American Cancer Society site: “Health Risks of Secondhand Smoke” |  | Google = GA = Siri |
| Q21:  What are the dangers to infants, children and unborn babies of smoking | 0 | C | Google information box gives info about SIDS and heart defects with smoking in pregnancy. Link is to: https://www.babycenter.com/0_how-smoking-during-pregnancy-affects-you-and-your-baby_1405720.bc the baby center’s article on smoking in pregnancy. | 2 | 0 | C | Speaks “according to baby center…” and provides information about SIDS and heart defects being more likely. Provides link to: https://www.babycenter.com/0_how-smoking-during-pregnancy-affects-you-and-your-baby_1405720.bc the baby center’s article on smoking in pregnancy. | 1 | 0 | C | [NOTE couldn't understand “unborn” 3 times. So the result is “unbalanced babies”]  Says, ‘here’s what I found on the web…’  Provides link to: https://www.babycenter.com/0_how-smoking-during-pregnancy-affects-you-and-your-baby_1405720.bc the baby center’s article on smoking in pregnancy. | Google = GA > Siri |  |
| Q21 | 0 | C | Google information box gives info about SIDS and heart defects with smoking in pregnancy. Link is to: https://www.babycenter.com/0_how-smoking-during-pregnancy-affects-you-and-your-baby_1405720.bc the baby center’s article on smoking in pregnancy. (it does cite various doctors but no medical review for the article) | 2 | 1 | C | Speaks “according to baby center…” and provides information about SIDS and heart defects being more likely. Provides link to: https://www.babycenter.com/0_how-smoking-during-pregnancy-affects-you-and-your-baby_1405720.bc the baby center’s article on smoking in pregnancy. | 2 | 0 | A | Links to the healthychildren.org site from the American Academy of Pediatrics: https://www.healthychildren.org/English/health-issues/conditions/tobacco/Pages/Dangers-of-Secondhand-Smoke.aspx |  | Siri > Google = GA |
| Q22. What is nicotine replacement therapy | 1 | B | Google information box explains what it is with a photo. The link provided is to Wikipedia entry on ‘NRT’ | 2 | 1 | B | Google information box on NRT is read aloud. The link to Wikipedia entry on NRT is given. | 1 | 0 | B | Says ‘here is what I found’, presents a short text summary and the link is to Wikipedia on NRT. | Google > GA = Siri |  |
| Q22 | 1 | B | Google information box explains what it is with a photo. The link provided is to Wikipedia entry on ‘NRT’ | 2 | 1 | B | Google information box on NRT is read aloud. The link to Wikipedia entry on NRT is given. | 2 | 0 | B | Says ‘here is what I found’, presents a short text summary and the link is to Wikipedia on NRT. |  | Google = GA = Siri |
| Q23. What are the different types of nicotine replacement therapy available | 1 | A | First link is American Cancer Society page on ‘NRT for quitting tobacco’: https://www.cancer.org/healthy/stay-away-from-tobacco/guide-quitting-smoking/nicotine-replacement-therapy.html | 0 | 1 | A | Doesn’t speak. First link is to cancer.org (ACS) on ‘NRT for quitting tobacco’ as for Google.  https://www.cancer.org/healthy/stay-away-from-tobacco/guide-quitting-smoking/nicotine-replacement-therapy.html | 1 | 2 | A | Says “Here’s what I found on the web for…” first link is to American Cancer Society page on ‘NRT for quitting tobacco’: https://www.cancer.org/healthy/stay-away-from-tobacco/guide-quitting-smoking/nicotine-replacement-therapy.html | Google = GA = Siri |  |
| Q23 | 1 | A | First link is American Cancer Society page on ‘NRT for quitting tobacco’: https://www.cancer.org/healthy/stay-away-from-tobacco/guide-quitting-smoking/nicotine-replacement-therapy.html | 0 | 2 | B | An informative patient information site that has a doctor as a peer reviewer: https://patient.info/health/nicotine-replacement-therapy | 2 | 0 | A | Says “Here’s what I found on the web for…” first link is to American Cancer Society page on ‘NRT for quitting tobacco’: https://www.cancer.org/healthy/stay-away-from-tobacco/guide-quitting-smoking/nicotine-replacement-therapy.html |  | Google = Siri > GA |
| Q24. Can anyone use nicotine replacement therapy | 1 | A | Google information box says many people can quit using NRT, but details the success and failure rather than who can use. Link then goes to American Cancer Society page on NRT for quitting (as above): https://www.cancer.org/healthy/stay-away-from-tobacco/guide-quitting-smoking/nicotine-replacement-therapy.html which details ‘who should and who should not use NRT’ | 3 | 0 | A | Speaks a Google information box about NRT, but details the success and failure rather than who can use. Link then goes to American Cancer Society page on NRT for quitting (as above): https://www.cancer.org/healthy/stay-away-from-tobacco/guide-quitting-smoking/nicotine-replacement-therapy.html which details ‘who should not use NRT’ | 1 | 1 | A | Says “here’s what I found on the web for…” there is *a Google information box which states that some people may not be able to use patched, inhalers, etc.* The first link is to American Cancer Society page on NRT for quitting (as above): https://www.cancer.org/healthy/stay-away-from-tobacco/guide-quitting-smoking/nicotine-replacement-therapy.html which details ‘who should not use NRT’ | Google = GA = Siri |  |
| Q24 | 0 | A | Google information box says many people can quit using NRT, but details the success and failure rather than who can use. Link then goes to American Cancer Society page on NRT for quitting (as above): https://www.cancer.org/healthy/stay-away-from-tobacco/guide-quitting-smoking/nicotine-replacement-therapy.html which details ‘who should and who should not use NRT’ | 3 | 0 | A | Google information box says many people can quit using NRT, but details the success and failure rather than who can use. Link then goes to American Cancer Society page on NRT for quitting (as above): https://www.cancer.org/healthy/stay-away-from-tobacco/guide-quitting-smoking/nicotine-replacement-therapy.html which details ‘who should and who should not use NRT’ | 2 | 0 | A | Links to a guide for health professionals by the NZ Ministry of Health: https://www.health.govt.nz/system/files/documents/publications/guide-to-prescribing-nicotine-replacement-therapy-nrtv2.pdf (possibly a bit complex for members of the public to understand) |  | Google = GA > Siri |
| Q25. Are nicotine replacement therapy products just as dangerous as smoking cigarettes | No | A | First link is to the same ACS page on NRT as above. This does discuss long-term dependence and nicotine overdose but NOT so much about danger compared to smoking. | 0 | No | A | First link is to the same ACS page on NRT as above. This does discuss long-term dependence and nicotine overdose but NOT so much about danger compared to smoking. | 1 | 1 | A | Says ‘here’s what I found on the web for…’ The first link is to American Cancer Society page on NRT for quitting (as above): https://www.cancer.org/healthy/stay-away-from-tobacco/guide-quitting-smoking/nicotine-replacement-therapy.html  This does discuss long-term dependence and nicotine overdose but NOT so much about danger compared to smoking. | Google = GA = Siri |  |
| Q25 | 0 | A | First link is to the same ACS page on NRT as above. This does discuss long-term dependence and nicotine overdose but NOT so much about danger compared to smoking. | 0 | 0 | A | First link is to the same ACS page on NRT as above. This does discuss long-term dependence and nicotine overdose but NOT so much about danger compared to smoking. | 2 | 0 | A | Says ‘here’s what I found on the web for…’ The first link is to American Cancer Society page on NRT for quitting (as above): https://www.cancer.org/healthy/stay-away-from-tobacco/guide-quitting-smoking/nicotine-replacement-therapy.html  This does discuss long-term dependence and nicotine overdose but NOT so much about danger compared to smoking. |  | Google = GA = Siri |
| Q26. Where can I get nicotine replacement therapy | 1 | A | Google information box says that you can buy it without a prescription. The link goes to Medline Plus medical encyclopaedia entry on NRT. It says what you can buy without a prescription but not where. https://medlineplus.gov/ency/article/007438.htm (MedlinePlus if from the US National Library of Medicine) | 3 | 1 | B | Reads aloud a Google information box says that you can buy it without a prescription. The first link is to WebMD ‘NRT for quitting tobacco’. This gives information that you can buy NRT without a prescription if you are over 18. | 1 | 0 | Fail | Says “I didn’t find any matching counseling services. Suggests (by text) that I try ‘Wikipedia therapy’ or ‘directions to a counseling service’ | Google = GA > Siri (fail) |  |
| Q26 | 1 | A | Google information box says that you can buy it without a prescription. The link goes to Medline Plus medical encyclopaedia entry on NRT. It says what you can buy without a prescription but not where. https://medlineplus.gov/ency/article/007438.htm (MedlinePlus if from the US National Library of Medicine) | 2 | 1 | A | Google information box says that you can buy it without a prescription. The link goes to Medline Plus medical encyclopaedia entry on NRT. It says what you can buy without a prescription but not where. https://medlineplus.gov/ency/article/007438.htm (MedlinePlus if from the US National Library of Medicine) | 1 | 0 | Fail | Says: “The only possibility I found is Amesbury Counselling Services on Grey Street in Palmerston North Central (a 2h 2m drive away). Actually the nearest source is just 800 metres away in a pharmacy or supermarket. |  | Google = GA > Siri (fail) |
| Q27. What is Zyban | 0 | A | First link is to Drugs.com entry on Zyban. Which has a tab linking to the FDA prescribing info https://www.drugs.com/zyban.html | 1 | 0 | B | Reads aloud the brief summary from Wikipedia entry on ‘bupropion’. States it is an antidepressant and smoking cessation aid. Gives the link to Wikipedia entry. | 1 | 0 | B | Says “all right, here’s what I got” then displays text explaining buproprion, stating it is an antidepressant and aid to smoking cessation, gives a diagram of its chemical structure. Gives link to Wikipedia entry on buproprion | Google > GA = Siri |  |
| Q27 | 0 | A | First link is to Drugs.com entry on Zyban. Which has a tab linking to the FDA prescribing info https://www.drugs.com/zyban.html | 1 | 0 | B | Links to the Wikipedia entry for bupropion. | 2 | 0 | B | As per the GA search (Wikipedia) |  | Google > GA = Siri |
| Q28. What is Champix | 0 | B | First link is to: https://www.champix.co.nz/what-is-champix Champix NZ commercial site. Which explains what it is. | 1 | 0 | B | Speaks ‘according to Wikipedia varenicline is a prescription medicine used to treat nicotine addiction’ – give the link to Wikipedia entry. | NA | NA | Fail | Could not understand ‘Champix’ on three attempts. Gave results for ‘champions’ | GA = Google > Siri (fail) |  |
| Q28 | 0 | B | First link is to: https://www.champix.co.nz/what-is-champix Champix NZ commercial site. Which explains what it is. | 1 | 0 | B | Speaks ‘according to Wikipedia varenicline is a prescription medicine used to treat nicotine addiction’ – give the link to Wikipedia entry. | NA | NA | Fail | Could not understand ‘Champix’ on three attempts. Gave results for ‘champions’ |  |  |
| Q29. Can I use e-cigarettes to help me quit | 0 | C | First link is to an e-cig blog/review site: https://ecig-reviews.net/using-e-cigarettes-quit-smoking/ It explains how to use e-cigs to quit, written by 2 ex-smokers. | 0 | 0 | C | First link is to an e-cig blog/review site: https://ecig-reviews.net/using-e-cigarettes-quit-smoking/ It explains how to use e-cigs to quit, written by 2 ex-smokers. | 1 | 0 | C | Says “here’s what I found on the web for “I use e-cigarettes…””  First link is to an e-cig blog/review site: https://ecig-reviews.net/using-e-cigarettes-quit-smoking/ It explains how to use e-cigs to quit, written by 2 ex-smokers. | Google = GA = Siri |  |
| Q29 | 0 | A | An informative NHS site: https://www.nhs.uk/smokefree/help-and-advice/e-cigarettes | 2 | 0 | A | An informative NHS site: https://www.nhs.uk/smokefree/help-and-advice/e-cigarettes | NA | NA | Fail | Says “Quit? Did you mean ‘goodbye’? |  | Google = GA > Siri (fail) |
| Q30. I'm not ready to quit, can I cut down gradually | No | A | First link is to a Cancer Institute of New South Wales ‘I can quit’ site. https://www.icanquit.com.au/quitting-methods/cut-down-to-quit that explains how to use the cut down method to quit. And that it can be effective. | 0 | No | Fail | First item is the LIVESTRONG MyQuit Coach App rated 4.5 at the app store. Gives ‘install’ button. | NA | NA | Fail | Says ‘was it something I said, I’ll go away if you say goodbye” | Google > GA (fail) > Siri (worse fail) |  |
| Q30 | 0 | A | First link is to a Cancer Institute of New South Wales ‘I can quit’ site. https://www.icanquit.com.au/quitting-methods/cut-down-to-quit that explains how to use the cut down method to quit. And that it can be effective. | 0 | 0 | Fail | First item is the LIVESTRONG MyQuit Coach App rated 4.5 at the app store. Gives ‘install’ button. | NA | NA | Fail | Says ‘was it something I said, I’ll go away if you say goodbye” |  | Google > GA=Fail, Siri (fail) |
| Q31. What are the dangers of smoking during pregnancy | No | A | Google information box about low birth weight and SIDS.  Link goes to the CDC: tobacco use and pregnancy site. https://www.cdc.gov/reproductivehealth/maternalinfanthealth/tobaccousepregnancy/index.htm | 3 | No | A | Reads aloud a summary of the CDC information on tobacco use in pregnancy. Gives a diagram. Talks about SIDS, infant death, low birth weight. Gives the link to CDC as did Google. | 1 | No | A | Says ‘ok I found this on the web for…’  Shows a Google box with diagram and information about SIDS, low birth weight etc. The first link is to CDC: tobacco use and pregnancy site. https://www.cdc.gov/reproductivehealth/maternalinfanthealth/tobaccousepregnancy/index.htm | Google = GA = Siri |  |
| Q31 | 0 | A | Google information box about low birth weight and SIDS.  Link goes to the CDC: tobacco use and pregnancy site. https://www.cdc.gov/reproductivehealth/maternalinfanthealth/tobaccousepregnancy/index.htm | 3 | 0 | A | Reads aloud a summary of the CDC information on tobacco use in pregnancy. Gives a diagram. Talks about SIDS, infant death, low birth weight. Gives the link to CDC as did Google. | 2 | 0 | B | Article reviewed by the BabyCenter Medical Advisory Board: https://www.babycenter.com/0_how-smoking-during-pregnancy-affects-you-and-your-baby_1405720.bc |  | Google = GA > Siri |
| Q32. Is it too late to stop smoking in pregnancy as isn't the damage already done | 1 | C | Newspaper article on ‘smoking in the first 4 months does not harm your baby’ Daily Mail Australia from 2008! http://www.dailymail.co.uk/health/article-514330/Smoking-months-pregnancy-does-harm-baby.html It’s a ‘researchers claim…’ article. | 0 | 0 | C | First link is to a 2007 Wiki question forum at ‘babycentre’ which is titled ‘I’ve been smoking and just now quit at 36 weeks will my baby still be ok. Various user supplied answers are given. | 1 | 1 | C | Says ‘Here’s what I found on the web for…’  First link is to a 2007 Wiki question forum at ‘babycentre’ which is titled ‘I’ve been smoking and just now quit at 36 weeks will my baby still be ok. Various user supplied answers are given. | Google > GA = Siri |  |
| Q32 | 0 | C | Queries by mothers answered by other mothers at a “babycenter community” site: https://www.babycenter.com/400_ive-been-smoking-and-just-now-quit-at-36-week-will-my-baby-s_965552_483.bc (no medical input) | 2 | 0 | C | Queries by mothers answered by other mothers at a “babycenter community” site: https://www.babycenter.com/400_ive-been-smoking-and-just-now-quit-at-36-week-will-my-baby-s_965552_483.bc (no medical input) | 0 | 0 | Fail | Says: “I’m afraid I don’t know the answer to that, Nick.” |  | Google = GA > Siri |
| Q33. My partner smokes so will this affect my baby | 0 | C | Fisher Price parenting topics article on dad smoking outside. http://www.fisher-price.com/en_SEA/playtime/parenting/articlesandadvice/articledetail.html?article=tcm:245-18787-16 mentions that smoking outdoors can still be harmful (though much less so) | 0 | 0 | C | Fisher Price parenting topics article on dad smoking outside. http://www.fisher-price.com/en_SEA/playtime/parenting/articlesandadvice/articledetail.html?article=tcm:245-18787-16 mentions that smoking outdoors can still be harmful (though much less so) | 1 | 0 | Fail | Says ‘OK, Matt, here’s what I found’  First link is the location map for ‘haven road store’ which is a tobacco shop in Nelson | Google = GA > Siri (fail) |  |
| Q33 | 0 | C | Fisher Price parenting topics article on dad smoking outside. http://www.fisher-price.com/en_SEA/playtime/parenting/articlesandadvice/articledetail.html?article=tcm:245-18787-16 mentions that smoking outdoors can still be harmful (though much less so) | 0 | 0 | C | Fisher Price parenting topics article on dad smoking outside. http://www.fisher-price.com/en_SEA/playtime/parenting/articlesandadvice/articledetail.html?article=tcm:245-18787-16 mentions that smoking outdoors can still be harmful (though much less so) | 2 | 0 | Fail | Says “One option I found is COSMIC on Cuba Street in Te Aro” (an e-cigarette retailer) |  | Google = GA > Siri |
| Q34. Can I use nicotine replacement therapy while pregnant | 0 | A | Google information box states that effective NRT may be used to help women quit while smoking. First link is to a PubMed article in the J Obst Gyn Canada. That concludes a combination of CBT and NRT is optimal during pregnancy. https://www.ncbi.nlm.nih.gov/pubmed/19772709 | 1 | 0 | A | Speaks ‘here’s a summary from NCI, ‘NRT in conjunction with CBT may offer an effective alternative to help pregnant women quit’ Gives same link as Google to PubMed article. | 1 | 1 | A | Says ‘here’s what I found on the web for…’  Google information box states that effective NRT may be used to help women quit while smoking. First link is to a PubMed article in the J Obst Gyn Canada. That concludes a combination of CBT and NRT is optimal during pregnancy. https://www.ncbi.nlm.nih.gov/pubmed/19772709 | Google = GA = Siri |  |
| Q34 | 0 | A | Google information box states that effective NRT may be used to help women quit while smoking. First link is to a PubMed article in the J Obst Gyn Canada. That concludes a combination of CBT and NRT is optimal during pregnancy. https://www.ncbi.nlm.nih.gov/pubmed/19772709 | 2 | 0 | A | Google information box states that effective NRT may be used to help women quit while smoking. First link is to a PubMed article in the J Obst Gyn Canada. That concludes a combination of CBT and NRT is optimal during pregnancy. https://www.ncbi.nlm.nih.gov/pubmed/19772709 | 2 | 0 | A | Google information box states that effective NRT may be used to help women quit while smoking. First link is to a PubMed article in the J Obst Gyn Canada. That concludes a combination of CBT and NRT is optimal during pregnancy. https://www.ncbi.nlm.nih.gov/pubmed/19772709 |  | Google = GA = Siri |
| Q35. What specialist support is available for pregnant smokers | 0 | A | First link is to NHS page on pregnancy and smoking: https://www.nhs.uk/smokefree/why-quit/smoking-in-pregnancy It lists and gives links to support services. | 0 | 0 | A | Gives link to NZ Ministry of health ‘services and support during pregnancy’ mostly about midwifery and maternity care, but gives the phone number for Healthline. [Doesn’t really answer the Q] | 1 | 0 | A | Says ‘here’s what I found on the web…’  First link is to NHS page on pregnancy and smoking: https://www.nhs.uk/smokefree/why-quit/smoking-in-pregnancy It lists and gives links to support services. | GA > Google = Siri |  |
| Q35 | 1 | A | Links to a full free text journal article on a survey of smoking cessation support for pregnant women in England: https://bmchealthservres.biomedcentral.com/articles/10.1186/1472-6963-14-107 | 0 | 0 | A | Gives link to NZ Ministry of health ‘services and support during pregnancy’ mostly about midwifery and maternity care, but gives the phone number for Healthline. (doesn’t answer the question directly but does cover general services that would probably still provide such support) | 2 | 0 | A | Links to a full free text journal article on a survey of smoking cessation support for pregnant women in England: https://bmchealthservres.biomedcentral.com/articles/10.1186/1472-6963-14-107 |  | Google = Siri > GA |
| Q36. Show me a video about smoking and asthma | 0 | A | Links to YouTube 45 second video of a paediatric immunologist/allergist explaining. https://www.youtube.com/watch?v=h3JdWWrgdzA | 1 | 0 | A | Says “Here are some matching videos” Links to YouTube 45 second video of a paediatric immunologist/allergist explaining. https://www.youtube.com/watch?v=h3JdWWrgdzA | NA | NA | Fail | Says ‘sorry I can only search by topic for movies’ | Google = GA > Siri (fail) |  |
| Q36 | 0 | A | Links to the YouTube CDC tips: https://www.youtube.com/watch?v=3eUOjSTZMIE | 1 | 0 | A | Links to the YouTube CDC tips: https://www.youtube.com/watch?v=3eUOjSTZMIE | 2 | 0 | Fail | Says ‘sorry I can only search by topic for movies’ |  | GA = Google > Siri |
| Q37. Show me a video about smoking and Buerger’s disease | 1 | A | Links to a youtube video from the CDC called ‘Tips from former smokers buergers disease ad’ https://www.youtube.com/watch?v=-WrWwUsKKN8 explains and shows the damage smoking can do. | 1 | 0 | A | Says “Here are some matching videos” Links to a youtube video from the CDC called ‘Tips from former smokers buergers disease ad’ https://www.youtube.com/watch?v=-WrWwUsKKN8 explains and shows the damage smoking can do. | NA | NA | Fail | Says ‘sorry I can only search by topic for movies’ | Google = GA > Siri (fail) |  |
| Q37 | 0 | A | Links to a youtube video from the CDC called ‘Tips from former smokers buergers disease ad’ https://www.youtube.com/watch?v=-WrWwUsKKN8 explains and shows the damage smoking can do. | 1 | 0 | A | Links to a youtube video from the CDC called ‘Tips from former smokers buergers disease ad’ https://www.youtube.com/watch?v=-WrWwUsKKN8 explains and shows the damage smoking can do. | 2 | 0 | Fail | Says ‘sorry I can only search by topic for movies’ |  | GA = Google > Siri |
| Q38. Show me a video about smoking and cancer | 0 | A | Links to a youtube video with 500k hits. It appears to be a public health advertisement https://www.youtube.com/watch?v=zcaeSKiZ37Y 30 seconds long and explains that one damaged cell can cause lung cancer. Every cigarette is doing damage. | 1 | 0 | A | Says “here are some matching videos” Links to a youtube video with 500k hits. It appears to be a public health advertisement https://www.youtube.com/watch?v=zcaeSKiZ37Y 30 seconds long and explains that one damaged cell can cause lung cancer. Every cigarette is doing damage. | NA | NA | Fail | Says ‘sorry I can only search by topic for movies’ | Google = GA > Siri (fail) |  |
| Q38 | 0 | B | An informative video on YouTube from Nucleus Medical Media: https://www.youtube.com/watch?v=gwuwrRK-I2Y  9 minutes, 560k views. The Review Board has certified doctors | 1 | 1 | A | Says “here are some matching videos” Links to a youtube video with 500k hits. It appears to be a public health advertisement https://www.youtube.com/watch?v=zcaeSKiZ37Y 30 seconds long and explains that one damaged cell can cause lung cancer. Every cigarette is doing damage.. This is part of the “Every Cigarette is Doing You Damage” campaign designed by Australian Health Authorities (and also run in NZ after modifications). | 2 | 0 | Fail | Says ‘sorry I can only search by topic for movies’ |  | GA > Google > Siri (fail) |
| Q39. Show me a video about smoking and chronic lung disease | 0 | B | Youtube video of ‘Understanding COPD#1’ https://www.youtube.com/watch?v=aktIMBQSXMo 3min 13 sec, 600K+ views. Made by ‘ilumistream health’ gives good general information. Interviews with doctors. | 1 | 0 | B | Youtube video of ‘Understanding COPD#1’ https://www.youtube.com/watch?v=aktIMBQSXMo 3min 13 sec, 600K+ views. Made by ‘ilumistream health’ gives good general information. Interviews with doctors. | NA | NA | Fail | Says ‘sorry I can only search by topic for movies’ | Google = GA > Siri (fail) |  |
| Q39 | 0 | B | Youtube video of ‘Understanding COPD#1’ https://www.youtube.com/watch?v=aktIMBQSXMo 3min 13 sec, 600K+ views. Made by ‘ilumistream health’ gives good general information. Interviews with doctors. | 1 | 0 | B | Youtube video of ‘Understanding COPD#1’ https://www.youtube.com/watch?v=aktIMBQSXMo 3min 13 sec, 600K+ views. Made by ‘ilumistream health’ gives good general information. Interviews with doctors. | 2 | 0 | Fail | Says ‘sorry I can only search by topic for movies’ |  | GA = Google > Siri (fail) |
| Q40. Show me a video about smoking and diabetes | 0 | A | CDC channel video on youtube, tips from former smokers, ‘Bill’s Ad: smoking and diabetes’ | 0 | 0 | A | CDC channel video on youtube, tips from former smokers, ‘Bill’s Ad: smoking and diabetes’ | NA | NA | Fail | Says ‘sorry I can only search by topic for movies’ | Google = GA > Siri |  |
| Q40 | 0 | B | YouTube video from dLife (a media company), 13k views, interviews with doctors: https://www.youtube.com/watch?v=BYa-Yk0uJIY | 0 | 0 | A | CDC channel video on youtube, tips from former smokers, ‘Bill’s Ad: smoking and diabetes’ | 2 | 0 | Fail | Says ‘sorry I can only search by topic for movies’ |  | GA > Google > Siri (fail) |
| Q41. Show me a video about smoking and gum disease | 0 | B | First video is a youtube video 68k views about periodontal disease. Uploaded by a dentist, there is a link to his website below the video. https://www.youtube.com/watch?v=If8xQDM-YS4 | 1 | 0 | B | Says ‘here are some matching videos’  First video is a youtube video 68k views about periodontal disease. Uploaded by a dentist, there is a link to his website below the video. https://www.youtube.com/watch?v=If8xQDM-YS4 | NA | NA | Fail | Says ‘sorry I can only search by topic for movies’ | Google = GA > Siri (fail) |  |
| Q41 | 0 | B | First video is a youtube video 68k views about periodontal disease. Uploaded by a dentist, there is a link to his website below the video. https://www.youtube.com/watch?v=If8xQDM-YS4 | 0 | 1 | B | Says ‘here are some matching videos’  First video is a youtube video 68k views about periodontal disease. Uploaded by a dentist, there is a link to his website below the video. https://www.youtube.com/watch?v=If8xQDM-YS4 | 2 | 0 | Fail | Says ‘sorry I can only search by topic for movies’ |  | Google = GA > Siri (fail) |
| Q42 Show me a video about smoking and heart disease | 0 | B | First link is a youtube video ‘smoking causes cancer, heart disease, emphysema’ https://www.youtube.com/watch?v=gwuwrRK-I2Y with 583k views. By nucleus medical media. | 1 | 0 | B | Says ‘here are some matching videos’  First link is a youtube video ‘smoking causes cancer, heart disease, emphysema’ https://www.youtube.com/watch?v=gwuwrRK-I2Y with 583k views. By nucleus medical media. | NA | NA | Fail | Says ‘sorry I can only search by topic for movies’ | Google = GA > Siri (fail) |  |
| Q42 | 0 | B | First link is a youtube video ‘smoking causes cancer, heart disease, emphysema’ https://www.youtube.com/watch?v=gwuwrRK-I2Y with 583k views. By nucleus medical media. | 1 | 2 | B | Says ‘here are some matching videos’  First link is a youtube video ‘smoking causes cancer, heart disease, emphysema’ https://www.youtube.com/watch?v=gwuwrRK-I2Y with 583k views. By nucleus medical media. | 2 | 0 | Fail | Says ‘sorry I can only search by topic for movies’ |  | Google = GA > Siri (fail) |
| Q43. Show me a video about smoking and stroke | 0 | A | First video is a youtube video from the Australian national quit smoking campaign about smoking and stroke, 80k views: https://www.youtube.com/watch?v=ghbc2Rlqu0g | 1 | 0 | A | Says ‘here are some matching videos’  First video is a youtube video from the Australian national quit smoking campaign about smoking and stroke, 80k views: https://www.youtube.com/watch?v=ghbc2Rlqu0g | NA | NA | Fail | Says ‘sorry I can only search by topic for movies’ | Google = GA > Siri (fail) |  |
| Q43 | 0 | B | First link is a youtube video ‘smoking causes cancer, heart disease, emphysema’ https://www.youtube.com/watch?v=gwuwrRK-I2Y with 583k views. By nucleus medical media. | 1 | 0 | B | First link is a youtube video ‘smoking causes cancer, heart disease, emphysema’ https://www.youtube.com/watch?v=gwuwrRK-I2Y with 583k views. By nucleus medical media. | 2 | 0 | Fail | Says ‘sorry I can only search by topic for movies’ |  | Google = GA > Siri (fail) |
| Q44. Show me a video about smoking and HIV | 0 | A | First video is a youtube video ‘conversations with AIDS.gov Dr Jonathon Mermin (CDC) on Smoking and HIV’ The interviewees discuss research literature and evidence. https://www.youtube.com/watch?v=KyRcGSx1T1s | 1 | 0 | A | Says ‘here are some matching videos’  First video is a youtube video ‘conversations with AIDS.gov Dr Jonathon Mermin on Smoking and HIV’ The interviewees discuss research literature and evidence. https://www.youtube.com/watch?v=KyRcGSx1T1s | NA | NA | Fail | Says ‘sorry I can only search by topic for movies’ | Google = GA > Siri (fail) |  |
| Q44 | 0 | A | First video is a youtube video ‘conversations with AIDS.gov Dr Jonathon Mermin (CDC) on Smoking and HIV’ The interviewees discuss research literature and evidence. https://www.youtube.com/watch?v=KyRcGSx1T1s | 0 | 0 | A | Says ‘here are some matching videos’  First video is a youtube video ‘conversations with AIDS.gov Dr Jonathon Mermin on Smoking and HIV’ The interviewees discuss research literature and evidence. https://www.youtube.com/watch?v=KyRcGSx1T1s | 2 | 0 | Fail | Says ‘sorry I can only search by topic for movies’ |  | Google = GA > Siri (fail) |
| Q45. Show me a video about smoking and depression | 0 | C | First video is a youtube video about ‘bipolar depression and smoking weed’ https://www.youtube.com/watch?v=h58IMeitYZU 9k views. 32 rambling minutes of a guy smoking weed. | 1 | 0 | C | Says ‘here are some matching videos’  First video is a youtube video about ‘bipolar depression and smoking weed’ https://www.youtube.com/watch?v=h58IMeitYZU 9k views. 32 rambling minutes of a guy smoking weed. | NA | NA | Fail | Says ‘sorry I can only search by topic for movies’ | Google = GA > Siri (fail) |  |
| Q45 | 0 | A | University centre with a doctor talking in a YouTube video (3k views): https://www.youtube.com/watch?v=RedXENL8BpY | 1 | 1 | C | Says ‘here are some matching videos’  First video is a youtube video about ‘bipolar depression and smoking weed’ https://www.youtube.com/watch?v=h58IMeitYZU 9k views. 32 rambling minutes of a guy smoking weed. | 2 | 0 | Fail | Says ‘sorry I can only search by topic for movies’ |  | Google = GA > Siri (fail) |
| Q46. Show me a video about smoking and anxiety | 0 | B | First video is a youtube video ‘smoking cigarettes gives me anxiety, what can I do?’ https://www.youtube.com/watch?v=CqGBJJWZvZ4 <1k views, posted by ‘Anxiety Boss’ it's a 2min self-help talk about overcoming anxiety around smoking.  * “The AnxietyBoss Channel is all about anxiety, presented by experts in anxiety.” | 1 | 0 | C | Says ‘here are some matching videos’  First link is to a youtube video called ‘smoking causes anxiety attacks’  9k views  Matt from iquitsmoking.com speaks about his experience as a smoker and about his anxiety attacks. He says smoking causes stress. | NA | NA | Fail | Says ‘sorry I can only search by topic for movies’ | Google > GA > Siri (fail) |  |
| Q46 | 0 | C | From iQuit-Smoking.Com smoking on anxiety and smoking (9k views): https://www.youtube.com/watch?v=PdPbVGjRB8U | 0 | 0 | C | From iQuit-Smoking.Com smoking on anxiety and smoking (9k views): https://www.youtube.com/watch?v=PdPbVGjRB8U | 2 | 0 | Fail | Says ‘sorry I can only search by topic for movies’ |  | Google = GA > Siri (fail) |
| Q47. Show me a video about smoking and pregnancy | 0 | C | First video is youtube and called ‘**4D ultrasound scans show what smoking does to an unborn baby during pregnancy** https://www.youtube.com/watch?v=aRULLIckHd0 it shows scan images, and text about harm to unborn baby, and recommendations from doctors. Posted by ‘Amazing world news’ | 0 | 0 | C | Doesn't say anything. First video is youtube and called ‘**4D ultrasound scans show what smoking does to an unborn baby during pregnancy** https://www.youtube.com/watch?v=aRULLIckHd0 it shows scan images, and text about harm to unborn baby, and recommendations from doctors. Posted by ‘Amazing world news’ | NA | NA | Fail | Says ‘sorry I can only search by topic for movies’ | Google = GA > Siri (fail) |  |
| Q47 | 0 | C | First video is youtube and called ‘**4D ultrasound scans show what smoking does to an unborn baby during pregnancy** https://www.youtube.com/watch?v=aRULLIckHd0 it shows scan images, and text about harm to unborn baby, and recommendations from doctors. Posted by ‘Amazing world news’ | 0 | 0 | C | Doesn't say anything. First video is youtube and called ‘**4D ultrasound scans show what smoking does to an unborn baby during pregnancy** https://www.youtube.com/watch?v=aRULLIckHd0 it shows scan images, and text about harm to unborn baby, and recommendations from doctors. Posted by ‘Amazing world news’ | 2 | 0 | Fail | Says ‘sorry I can only search by topic for movies’ |  | Google = GA > Siri |
| Q48. Show me a video about smoking and quitting | 0 | C | First video is youtube called ‘quitting smoking timeline’  https://www.youtube.com/watch?v=fLbQfMmrISE  Explains what happens to your body over time as you stop smoking. 1.5M views. Posted by ‘QuitSmokingCom’ | 0 | 0 | C | Doesn't say anything.  First video is youtube called ‘quitting smoking timeline’  https://www.youtube.com/watch?v=fLbQfMmrISE  Explains what happens to your body over time as you stop smoking. 1.5M views. Posted by ‘QuitSmokingCom’ | NA | NA | Fail | Says ‘sorry I can only search by topic for movies’ | Google = GA > Siri (fail) |  |
| Q48 | 0 | C | First video is youtube called ‘quitting smoking timeline’  https://www.youtube.com/watch?v=fLbQfMmrISE  Explains what happens to your body over time as you stop smoking. 1.5M views. Posted by ‘QuitSmokingCom’ | 0 | 0 | C | Doesn't say anything.  First video is youtube called ‘quitting smoking timeline’  https://www.youtube.com/watch?v=fLbQfMmrISE  Explains what happens to your body over time as you stop smoking. 1.5M views. Posted by ‘QuitSmokingCom’ | 2 | 0 | Fail | Says ‘sorry I can only search by topic for movies’ |  | Google = GA > Siri |
| Q49. Show me a video about smoking and blindness | 0 | A | First video is youtube: https://www.youtube.com/watch?v=MmKgm--EQUU from the CDC, Marlene’s story about macular degeneration. It’s an antismoking capaign ad. | 1 | 0 | A | Says ‘here are some matching videos’  First video is youtube: https://www.youtube.com/watch?v=MmKgm--EQUU from the CDC, Marlene’s story about macular degeneration. It’s an antismoking capaign ad. | NA | NA | Fail | Says ‘sorry I can only search by topic for movies’ | Google = GA > Siri (fail) |  |
| Q49 | 0 | A | First video is youtube: https://www.youtube.com/watch?v=MmKgm--EQUU from the CDC, Marlene’s story about macular degeneration. It’s an antismoking capaign ad. | 0 | 0 | A | Says ‘here are some matching videos’  First video is youtube: https://www.youtube.com/watch?v=MmKgm--EQUU from the CDC, Marlene’s story about macular degeneration. It’s an antismoking capaign ad. | 2 | 0 | Fail | Says ‘sorry I can only search by topic for movies’ |  | Google = GA > Siri |
| Q50. Show me a video about smoking and vision loss | 0 | A | First video is Youtube from the CDC, ‘Marlene’s vision loss’ https://www.youtube.com/watch?v=hT4QqyBP1tE another macular degeneration video. | 1 | 0 | A | Says ‘here are some matching videos’  First video is Youtube from the CDC, ‘Marlene’s vision loss’ https://www.youtube.com/watch?v=hT4QqyBP1tE another macular degeneration video. | NA | NA | Fail | Says ‘sorry I can only search by topic for movies’ | Google = GA > Siri (fail) |  |
| Q50 | 0 | A | First video is Youtube from the CDC, ‘Marlene’s vision loss’ https://www.youtube.com/watch?v=hT4QqyBP1tE another macular degeneration video. | 0 | 0 | A | Says ‘here are some matching videos’  First video is Youtube from the CDC, ‘Marlene’s vision loss’ https://www.youtube.com/watch?v=hT4QqyBP1tE another macular degeneration video. | 2 | 0 | Fail | Says ‘sorry I can only search by topic for movies’ |  | Google = GA > Siri |
| Q51. Show me a video about smoking and being lesbian | 0 | Fail | Youtube video is first showing two women breathing e-cig vapour from mouth to mouth. https://www.youtube.com/watch?v=qsXRrtKbDYM | 0 | 0 | Fail | Doesn’t say anything.  First video is ‘lesbian smoking fetish Smokey kisses’ on youtube  55k views shows two women smoking seductively. | NA | NA | Fail | Says ‘sorry I can only search by topic for movies’ | All Fail |  |
| Q51 | 0 | Fail | Youtube video is first showing two women breathing e-cig vapour from mouth to mouth. https://www.youtube.com/watch?v=qsXRrtKbDYM | 0 | 0 | Fail | Youtube video is first showing two women breathing e-cig vapour from mouth to mouth. https://www.youtube.com/watch?v=qsXRrtKbDYM | 2 | 0 | Fail | Says ‘sorry I can only search by topic for movies’ |  | All fail |
| Q52. Show me a video about smoking and being gay | 0 | Fail | First video is youtube, titled ‘new antismoking video tells teens its gay to smoke’  https://www.youtube.com/watch?v=82x9pzHkHK4 It’s a satirical news item from ‘The Onion’ | 1 | 0 | Fail | Says ‘here are some matching videos’  First video is youtube, titled ‘new antismoking video tells teens its gay to smoke’  https://www.youtube.com/watch?v=82x9pzHkHK4 It’s a satirical news item from ‘The Onion’ | NA | NA | Fail | Says ‘sorry I can only search by topic for movies’ | All Fail |  |
| Q52 | 0 | Fail | An article on Vice.com which has no useful information: https://www.vice.com/en_nz/article/9a4a9e/hold-on-can-smoking-weed-actually-make-you-gay-weedweek2017 | 0 | 0 | Fail | An article on Vice.com which has no useful information: https://www.vice.com/en_nz/article/9a4a9e/hold-on-can-smoking-weed-actually-make-you-gay-weedweek2017 | 2 | 0 | Fail | Says ‘sorry I can only search by topic for movies’ |  | All fail |
| Q53. Show me a YouTube video of a person with lung disease from smoking trying to breath | 0 | Fail | First video is youtube, https://www.youtube.com/watch?v=2nBPqSiLg5E titled ‘COPD, Chronic obstructive pulomanry disease’ it is mostly an animation explaining COPD. Posted by Nucleus Medical Media  *No video of live people breathing* | 1 | 0 | Fail | Says ‘here are some matching videos’  First video is youtube, https://www.youtube.com/watch?v=2nBPqSiLg5E titled ‘COPD, Chronic obstructive pulomanry disease’ it is mostly an animation explaining COPD. Posted by Nucleus Medical Media  *No video of live people breathing* | 1 | 0 | Fail | ‘Here are some videos of… I found on the web’  Frist video is youtube, ‘What causes COPD’ 41k views. Manipal Hospitals. Animations explain COPD, *No video of live people breathing.* | All Fail |  |
| Q53 | 0 | Fail | First video is youtube, https://www.youtube.com/watch?v=2nBPqSiLg5E titled ‘COPD, Chronic obstructive pulomanry disease’ it is mostly an animation explaining COPD. Posted by Nucleus Medical Media  *No video of live people breathing* | 1 | 0 | Fail | Says ‘here are some matching videos’  First video is youtube, https://www.youtube.com/watch?v=2nBPqSiLg5E titled ‘COPD, Chronic obstructive pulomanry disease’ it is mostly an animation explaining COPD. Posted by Nucleus Medical Media  *No video of live people breathing* | 1 | 0 | Fail | Goes to YouTube video “What is emphysema?” from Khan Academy (11 minute video; 156,000 views) American Association of Colleges of Nursing logo at the start. https://www.khanacademy.org/science/health-and-medicine/respiratory-system-diseases/emphysema/v/what-is-emphysema (is relatively technical - like a lecture). |  | All fail |
| Q54. Show me a YouTube video of the tar in cigarette smoke when breathing out into a tissue | 0 | Fail | First video is youtube video https://www.youtube.com/watch?v=l26f4f-V4jc ‘effects of smoking’ Doesn’t appear to show anything to do with a tissue or tar. 387k views | 1 | 0 | Fail | Says ‘here are some matching videos’  First video is youtube video https://www.youtube.com/watch?v=l26f4f-V4jc ‘effects of smoking’ Doesn’t appear to show anything to do with a tissue or tar. 387k views | 1 | 0 | Fail | Says ‘here are some videos of… that I found on the web’  [NB took 2 repetitions to get ‘tar’]  First video is Youtube, ‘smoking causes cancer, heart disease, emphesyma’ 580k views, Nucleus Medical Media, animations only. *No video of people breathing into tissue.* | All Fail |  |
| Q54 | 0 | Fail | First video is youtube video https://www.youtube.com/watch?v=l26f4f-V4jc ‘effects of smoking’ Doesn’t appear to show anything to do with a tissue or tar. 387k views | 1 | 0 | Fail | Says ‘here are some matching videos’  First video is youtube video https://www.youtube.com/watch?v=l26f4f-V4jc ‘effects of smoking’ Doesn’t appear to show anything to do with a tissue or tar. 387k views | 0 | 0 | Fail | Links to a very brief video on lung cancer development: https://www.youtube.com/watch?v=qQZu0CYj_lo but nil on any people breathing into a tissue |  | All fail |
| Q55. Show me a YouTube video of an anti-smoking TV commercial | 0 | A | First video is youtube, an antismoking ad from New Zealand https://www.youtube.com/watch?v=aiuxMiwvhZY  World SmokeFree Day campaign ad. | 1 | 0 | A | Says ‘here are some matching videos’  First video is youtube, an antismoking ad from New Zealand https://www.youtube.com/watch?v=aiuxMiwvhZY  World SmokeFree Day campaign ad. | 1 | 0 | Fail | Says ‘here are some videos of… that I found on the web’  First video is youtube, 60 year old ad, PROMOTING smoking. 494k views. | Google = GA > Siri (fail) |  |
| Q55 | 0 | A | Commercials from various countries eg, USA, US states, UK and Australia (produced as part of campaigns [eg, Truth Campaign], some authorised by the government, CDC): “Top 40: scariest anti-smoking commercials” https://www.youtube.com/watch?v=9kKN8_aa38A | 1 | 0 | A | Commercials from various countries eg, USA, US states, UK and Australia (produced as part of campaigns [eg, Truth Campaign], some authorised by the government, CDC): “Top 40: scariest anti-smoking commercials” https://www.youtube.com/watch?v=9kKN8_aa38A | 2 | 0 | Fail | Says ‘sorry I can only search by topic for movies’ |  | Google = GA > Siri (fail) |
| Q56. Show me a YouTube video of tobacco executives lying about nicotine not being addictive | 0 | NA | First video is youtube, looks to be a congressional inquiry into tobacco companies, and a senator is asking CEOs if they think nicotine is addictive. A range of CEOs answer No. https://www.youtube.com/watch?v=A6B1q22R438 | 0 | 0 | NA | Doesn’t say anything  First video is youtube, looks to be a congressional inquiry into tobacco companies, and a senator is asking CEOs if they think nicotine is addictive. A range of CEOs answer No. https://www.youtube.com/watch?v=A6B1q22R438 | 1 | 0 | NA | Says ‘here are some videos of… that I found on the web’  First video is youtube, looks to be a congressional inquiry into tobacco companies, and a senator is asking the CEO of philip morris if his product is addictive. CEO is saying basically no its not. | Google = GA = Siri |  |
| Q56 | 0 | NA | First video is youtube, looks to be a congressional inquiry into tobacco companies, and a senator is asking CEOs if they think nicotine is addictive. A range of CEOs answer No. https://www.youtube.com/watch?v=A6B1q22R438 | 0 | 0 | NA | Doesn’t say anything  First video is youtube, looks to be a congressional inquiry into tobacco companies, and a senator is asking CEOs if they think nicotine is addictive. A range of CEOs answer No. https://www.youtube.com/watch?v=A6B1q22R438 | 2 | 0 | NA | First video is youtube, looks to be a congressional inquiry into tobacco companies, and a senator is asking CEOs if they think nicotine is addictive. A range of CEOs answer No. https://www.youtube.com/watch?v=A6B1q22R438 |  | Google = GA = Siri |
| Q57. Show me a YouTube video on how to refill an e-cigarette tank | 0 | NA | First video is youtube ‘how to fill up my e-cigarette tank’ https://www.youtube.com/watch?v=wVsbX2NNxME simple, clear demonstration | 1 | 0 | NA | Says ‘here are some matching videos’  First video is youtube ‘how to fill up my e-cigarette tank’ https://www.youtube.com/watch?v=wVsbX2NNxME simple, clear demonstration | 1 | 0 | Fail | Says ‘sorry I can’t search what something is about but I can search by title, actors or directors and categories like horror or action | Google = GA > Siri (fail) |  |
| Q57 | 0 | NA | First video is youtube ‘how to fill up my e-cigarette tank’ https://www.youtube.com/watch?v=wVsbX2NNxME simple, clear demonstration | 1 | 0 | NA | Says ‘here are some matching videos’  First video is youtube ‘how to fill up my e-cigarette tank’ https://www.youtube.com/watch?v=wVsbX2NNxME simple, clear demonstration | 1 | 0 | NA | Goes to a video of how to fill a vape pen: “How To Vape - Filling Your Ego CE4 Clearomizer With E Liquid” (22k views) https://www.youtube.com/watch?v=jy6xbPITauM |  | Google = GA = Siri |
| Q58. Show me a picture of a smoker’s lungs | 0 | NA | First image is of a smokers lungs, and is hosted on this page: http://smokerslungs.net/page/3 | 1 | 0 | NA | Says ‘check out these pictures for a smokers lungs’  First image is black smokers lungs in a plastic container. | 1 | 0 | NA | Says ‘here are some images of a smokers lungs I found on the web’  First image is a graphic of a normal lung beside a black lung. | Google = GA > Siri (Siri’s wasn’t a photo) |  |
| Q58 | 0 | NA | A range of informative images shown with the first one linking through to an informative YouTube video:  https://www.youtube.com/watch?v=yskYG-EVlBY | 0 | 0 | NA | A range of informative images shown with the first one linking through to an informative YouTube video:  https://www.youtube.com/watch?v=yskYG-EVlBY | 1 | 0 | NA | Says ‘here is an image of a smokers lungs I found on the web’  First image is a graphic of a normal lung beside a black lung. |  | Google = GA > Siri |
| Q59. Show me a picture of lung cancer | 0 | B | First link takes you to ‘Medical News Today’ (newsletter reviewed by an MPH) site’s article on ‘lung cancer in pictures’  https://www.medicalnewstoday.com/articles/316538.php which has an image of an x-ray and pathology specimen. | 1 | 0 | Fail | Says ‘pictures of lung cancer’.  First image is a graphic comparing normal and smokers lungs. It does not really show lung cancer. | 1 | 0 | NA | Says ‘here are some images of lung cancer that I found on the web’  First picture is a cartoon-ish graphic of a red glow in transparent lungs. | Google > Siri > GA (fail) |  |
| Q59 | 0 | B | A range slideshow of informative images on WebMD: https://www.webmd.com/lung-cancer/ss/slideshow-lung-cancer-overview | 0 | 0 | B | A range slideshow of informative images on WebMD: https://www.webmd.com/lung-cancer/ss/slideshow-lung-cancer-overview | 1 | 0 | NA | Says ‘here are some images of lung cancer that I found on the web’  First picture is a cartoon-ish graphic of a red glow in transparent lungs. (on Bing) |  | Google = GA > Siri |
| Q60. Show me a picture of how smoking makes you look older. | 0 | C | First image is of an old man, and is hosted at Huffington Post http://www.huffingtonpost.com/2013/11/04/smoking-look-older-facial-aging-premature_n_4184292.html the article is called ‘photos: smoking makes you look older” but the page has a range of loading errors. | 1 | 0 | NA | Says ‘pictures of how smoking makes you look older’  First image is called ‘twin study of how smoking makes you look older’ and shows a pair of twins. | 1 | 0 | NA | Says ‘here are some imgages of how smoking… that I found on the web.  First image is: ‘smoking really does make you look older’ and is of a woman with wrinkles and lines. | GA > Siri > Google |  |
| Q60 | 0 | B | A range slideshow of informative images on WebMD: https://www.webmd.com/lung-cancer/ss/slideshow-lung-cancer-overview | 0 | 0 | B | A range slideshow of informative images on WebMD: https://www.webmd.com/lung-cancer/ss/slideshow-lung-cancer-overview | 1 | 0 | NA | Says ‘here are some imgages of how smoking… that I found on the web.  First image is: ‘smoking really does make you look older’ and is of a woman with wrinkles and lines. (on Bing) |  | Google = GA > Siri |
| Q61. Show me a picture of how smoking causes wrinkles | 0 | C | First image is of a wrinkly face with bad teeth. Image is hosted at business insider and the article is called ‘this is what smoking does to your face” http://www.businessinsider.com/this-is-what-smoking-does-to-your-face-2015-9?IR=T | 1 | 0 | NA | Says ‘check out these pictures for how smoking causes wrinkles’  First image is a half/half face of the before/after kind. Where make-up was used to show what someone could look like in the future. | 1 | 0 | NA | Says ‘here are some images of… that I found on the web’  First image is: a half/half face showing smoking vs non-smoking, includes wrinkles. | Google > GA = Siri |  |
| Q61 | 0 | C | First image is of a wrinkly face with bad teeth. Image is hosted at business insider and the article is called ‘this is what smoking does to your face” http://www.businessinsider.com/this-is-what-smoking-does-to-your-face-2015-9?IR=T | 1 | 1 | C | First image is of a wrinkly face with bad teeth. Image is hosted at business insider and the article is called ‘this is what smoking does to your face” http://www.businessinsider.com/this-is-what-smoking-does-to-your-face-2015-9?IR=T | 1 | 0 | NA | Says ‘here are some images of… that I found on the web’  First image is: a half/half face showing smoking vs non-smoking, includes wrinkles. |  | Google = GA = Siri |
| Q62. Show me a diagram of all the health problems that smoking can cause | 0 | A | First image is a diagram hosted by the CDC showing harm smoking can cause: https://www.cdc.gov/tobacco/data_statistics/fact_sheets/health_effects/effects_cig_smoking/index.htm | 1 | 0 | A | Says ‘here are some matching pictures’  First image is a diagram from a CDC fact sheet pointing out the harm smoking can do to children and adults. | 1 | 0 | A | Says ‘heres what I found on the web for…’  First link is to the CDC ‘health effects of cigarette smoking’.  Page includes an infographic. | Google = GA = Siri |  |
| Q62 | 0 | A | First image is a diagram hosted by the CDC showing harm smoking can cause: https://www.cdc.gov/tobacco/data_statistics/fact_sheets/health_effects/effects_cig_smoking/index.htm | 1 | 0 | A | First image is a diagram hosted by the CDC showing harm smoking can cause: https://www.cdc.gov/tobacco/data_statistics/fact_sheets/health_effects/effects_cig_smoking/index.htm | 1 | 0 | B | YouTube video by a Dr Mercola – but not a diagram: https://articles.mercola.com/smoking-side-effects.aspx |  | Google = GA > Siri |
| Q63. Find the best app for quitting smoking | 0 | B | Google information box and a link to ‘best apps to help you quit’ by healthline https://www.healthline.com/health/quit-smoking/top-iphone-android-apps several apps are reviewed by the authors. | 0 | 1 | B | Doesn’t say anything.  Just a link to ‘best apps to help you quit’ by healthline https://www.healthline.com/health/quit-smoking/top-iphone-android-apps several apps are reviewed by the authors. | 1 | 1 | C | Says ‘ok searching for quitting smoking on the app store’  First app is ‘Smoke Free’ with 4.6 star rating.  By David Crane (not the NHS one) | Google = GA > Siri |  |
| Q63 | 4 | B | Google information box and a link to ‘best apps to help you quit’ by healthline https://www.healthline.com/health/quit-smoking/top-iphone-android-apps several apps are reviewed by the authors. (noting that Healthline does have medical reviewers) | 0 | 3 | B | Doesn’t say anything.  Just a link to ‘best apps to help you quit’ by healthline https://www.healthline.com/health/quit-smoking/top-iphone-android-apps several apps are reviewed by the authors. | 1 | 0 | C | Says ‘ok searching for quitting smoking on the app store’  First app is ‘Smoke Free’ with 4.6 star rating.  By David Crane (not the NHS one) |  | Google = GA > Siri |
| Q64. Find an app for quitting smoking designed by health experts | 1 | Fail | First link goes to: https://www.quitgenius.com/ but there is a warning saying ‘your connection is not private’ and the page does not load. Second link goes to healthline as above: https://www.healthline.com/health/quit-smoking/top-iphone-android-apps | 0 | 0 | A | First app listed is ‘Stop-tobacco’ with not enough reviews for a rating. Developed by ‘Universite de Geneve’ | 1 | 0 | C | Says ‘ok searching for health smoking on the app store’  First app is: Smoke Free by David Crane (not the NHS one) | GA > Siri > Google (fail) |  |
| Q64 | 0 | B | First link goes to: https://www.quitgenius.com/ but there is a warning saying ‘your connection is not private’ and the page does not load. Second link goes to healthline as above: https://www.healthline.com/health/quit-smoking/top-iphone-android-apps (but the app download not tested on the laptop) | 0 | 2 | A | First app listed is ‘Stop-tobacco’ with not enough reviews for a rating. Developed by ‘Universite de Geneve’ | 1 | 1 | Fail | Only shows an App as part of an advertisement: “Subliminal Work – Positive Thinking Affirmations” |  | GA > Google > Siri (fail) |
| Q65. Find the best app for cutting down alcohol (For helping quit smoking). | 1 | B | First link goes to healthline https://www.healthline.com/health/addiction/top-alcoholism-iphone-android-apps where a range of alcoholism apps are reviewed by the authors. | 0 | 1 | B | Says nothing. First link goes to healthline https://www.healthline.com/health/addiction/top-alcoholism-iphone-android-apps where a range of alcoholism apps are reviewed by the authors. | 1 | 0 | Fail | Says ‘ok searching for cutting down alcohol on the app store’  Result “NO results ‘cutting down alcohol’ | Google = GA > Siri (fail) |  |
| Q65 | 1 | B | First link goes to healthline https://www.healthline.com/health/addiction/top-alcoholism-iphone-android-apps where a range of alcoholism apps are reviewed by the authors. (there is some focus on cutting down – but the focus on alcoholism may be off-putting to many people who don’t regard themselves as alcoholic) | 0 | 1 | B | Says nothing. First link goes to healthline https://www.healthline.com/health/addiction/top-alcoholism-iphone-android-apps where a range of alcoholism apps are reviewed by the authors. | 1 | 0 | Fail | The first app shown is on “Tai Chi Fundamentals” (which seems likely to be too indirect for most people in terms of the alcohol issue) |  | GA = Google > Siri (fail) |
| Q66. What is the Quitline phone number. | 1 | A | Google information box gives the NZ quitline number, Link goes to the NZ Ministry of health stop smoking page. | 2 | 1 | A | Speaks the NZ number and says according to the Ministry of Health. | 1 | 0 | Fail | Says ‘did I say something wrong…’ | Google = GA > Siri (fail) |  |
| Q66 | 1 | A | Google information box gives the NZ quitline number, Link goes to the NZ Ministry of health stop smoking page. | 2 | 1 | A | Speaks the NZ number and says according to the Ministry of Health. | 2 | 0 | Fail | Says ‘did I say something wrong…’ |  | GA = Google > Siri (fail) |
| Q67. Can I phone the Quitline now. (Testing for opening hours). | 0 | A | First link goes to https://quit.org.nz/ and the phone number is in the top right corner. The text reads ‘call us now’ but doesn’t give hours. | 0 | 0 | A | Says nothing. First link goes to https://quit.org.nz/ and the phone number is in the top right corner. The text reads ‘call us now’ but doesn’t give hours. | 1 | 0 | Fail | Says ‘did I say something wrong…’ | Google = GA > Siri (fail) |  |
| Q67 | 0 | A | First link goes to https://quit.org.nz/ and the phone number is in the top right corner. The text reads ‘call us now’ but doesn’t give hours. | 0 | 2 |  | Says nothing. First link goes to https://quit.org.nz/ and the phone number is in the top right corner. The text reads ‘call us now’ but doesn’t give hours. | 2 | 0 | Fail | Says “Was it something I said? I’ll go away if you say ‘goodbye’. |  | Google = GA > Siri (fail) |
| Q68. Where is the nearest family doctor | 0 | NA | Given directions the Reefton medical centre. This is indeed the nearest family doctor. 900m | 0 | 0 | NA | Given directions the Reefton medical centre. This is indeed the nearest family doctor. 900m | 1 | 0 | Fail | Says “I couldn’t find any matching family practice physicians” | Google = GA > Siri (fail) |  |
| Q68 | 0 | NA | Given directions to Karori Medical Centre. This is indeed the nearest family doctor at 800m away | 1 | 0 | NA | Given directions to Karori Medical Centre. This is indeed the nearest family doctor at 800m away | 1 | 0 | Fail | Says “I couldn’t find any matching family practice physicians” |  | Google = GA > Siri (fail) |
| Q69. Where is the nearest place to buy nicotine patches | 2 | NA | First link is http://www.habitrol.com/where-to-buy-nicotine-patches  A US product, but the page gives you options for buying online. | 0 | 2 | NA | First link is http://www.habitrol.com/where-to-buy-nicotine-patches  A US product, but the page gives you options for buying online. | 0 | 0 | Fail | Text only says ‘what kind of businesses are you looking for?’ | Google = GA > Siri (fail) |  |
| Q69 | 2 | NA | First link is http://www.habitrol.com/where-to-buy-nicotine-patches  A US product, but the page gives you options for buying online. | 0 | 2 | NA | First link is http://www.habitrol.com/where-to-buy-nicotine-patches  A US product, but the page gives you options for buying online. | 1 | 0 | Fail | Text only says ‘what kind of businesses are you looking for?’ |  | Google = GA > Siri (fail) |
| Q70. Where is the nearest place to buy nicotine gum | 2 | NA | First link is to a site for Nicorette, ‘where to buy’ https://www.nicorette.co.nz/where-to-buy NZ local. It says you can buy nicorette from your local pharmacy. | 0 | 2 | NA | First link is to a site for Nicorette, ‘where to buy’ https://www.nicorette.co.nz/where-to-buy NZ local. It says you can buy nicorette from your local pharmacy. | 1 | 0 | Fail | Says ‘what kind of businesses are you looking for?’ | Google = GA > Siri (Fail) |  |
| Q70 | 2 | NA | First link is to a site for Nicorette, ‘where to buy’ https://www.nicorette.co.nz/where-to-buy NZ local. It says you can buy nicorette from your local pharmacy. | 0 | 2 | Fail | A university site on NRT but it does not detail the locations for purchase: http://www.otago.ac.nz/wellington/research/otago064211.html | 1 | 0 | Fail | Says ‘what kind of businesses are you looking for?’ |  | Google > GA=Fail, Siri (fail) |
| Q71. Where is the nearest place to buy e-cigarettes | 0 | Fail | First link is ‘e-cig store near me’ https://www.njoy.com/find-a-store need to confirm I’m over 18 to continue. There is then a store locator for the US. | 0 | 0 | Fail | First link is ‘e-cig store near me’ https://www.njoy.com/find-a-store need to confirm I’m over 18 to continue. There is then a store locator for the US. | 1 | 0 | Fail | Says ‘I couldn’t find any matching places’ | All Fail |  |
| Q71 | 2 | NA | Finds one 4.2 km away. http://www.shosha.co.nz/stores (but there is another closer one at 4.1km that is further down the list of the search) | 0 | 0 | NA | Finds one 4.2 km away. http://www.shosha.co.nz/stores (but there is another closer one at 4.1km that is further down the list of the search) | 1 | 0 | NA | Finds the Cosmic Store in Te Aro which is 6.9km away |  | Google = GA > Siri (fail) |
| Q72. Where is the nearest café or restaurant with outdoor smokefree dining | 0 | Fail | First link is a news item on Stuff about smokefree dining trials in cafes 2016: http://www.stuff.co.nz/national/health/85758915/City-cafes-and-restaurants-trial-smokefree-outdoor-dining  It says ’20 in Christchurch’ but doesn't give a list of locations. | 0 | 0 | Fail | First link is a news item on Stuff about smokefree dining trials in cafes 2016: http://www.stuff.co.nz/national/health/85758915/City-cafes-and-restaurants-trial-smokefree-outdoor-dining  It says ’20 in Christchurch’ but doesn't give a list of locations. | 1 | 0 | Fail | Says ‘I didn’t find any matching restaurants’ | All Fail (no specific locations given) |  |
| Q72 | 0 | Fail | First link is a news item on Stuff about smokefree dining trials in cafes 2016: http://www.stuff.co.nz/national/health/85758915/City-cafes-and-restaurants-trial-smokefree-outdoor-dining  It says ’20 in Christchurch’ but doesn't give a list of locations. | 0 | 0 | Fail | First link is a news item on Stuff about smokefree dining trials in cafes 2016: http://www.stuff.co.nz/national/health/85758915/City-cafes-and-restaurants-trial-smokefree-outdoor-dining  It says ’20 in Christchurch’ but doesn't give a list of locations. | 1 | 0 | Fail | Says ‘I didn’t find any matching restaurants’ |  | All fail |
| Q73. Where is the nearest bar or pub with outdoor smokefree drinking | 0 | Fail | First link is to a news item on stuff titled ‘hospitality industry blasts smoking ban bid” from 2015: http://www.stuff.co.nz/business/70365797/hospitality-industry-blasts-smoking-ban-bid It is not about locations | 0 | 0 | Fail | First link is to a news item on stuff titled ‘hospitality industry blasts smoking ban bid” from 2015: http://www.stuff.co.nz/business/70365797/hospitality-industry-blasts-smoking-ban-bid It is not about locations | 1 | 0 | NA | Says ‘ok check it out’ and gives directions to Liquid Bar in Nelson. No info whether its smokefree. | Siri > GA (fail) = Google (fail) |  |
| Q73 | 0 | NA | Finds what claims to be the only Wellington bar with a smokefree outdoor setting: http://www.goldingsfreedive.co.nz/page/home.aspx | 0 | 1 | Fail | First link is to a news item on stuff titled ‘hospitality industry blasts smoking ban bid” from 2015: http://www.stuff.co.nz/business/70365797/hospitality-industry-blasts-smoking-ban-bid It is not about locations | 1 | 0 | Fail | Goes to blackdogbrewery.co.nz but no evidence this has smokefree outdoors |  | Google > GA=Fail, Siri (fail) |
| Q74. Where is the nearest smokefree childrens’ playground | 0 | A | First link is to a CDHB page https://www.cph.co.nz/your-health/smokefree-community-spaces/ about smokefree community it states that “All 12 Councils in the Canterbury West Coast region now have policies for smokefree outdoor areas in their parks, playgrounds and sports grounds“ *Does not give info on the nearest one though.* | 0 | 0 | Fail (not local) | First link is to a PDF listing smoke-free playground locations in South Australia. | 1 | 0 | Fail | Says ‘what kind of place are you looking for, tobacconists or playgrounds?’  Clicking on playgrounds and Siri says ‘I couldn’t find any matching playgrounds’ | Google > GA (fail) > Siri (worse fail) |  |
| Q74 | 0 | NA | Provides a link to all Wellington playgrounds – which are all described in this website as being smokefree: https://wellington.govt.nz/recreation/stuff-for-kids/play-areas/facilities | 0 | 1 | Fail | First link is a NZ Cancer Society website on “Smokefree Outdoor Areas” but with no specifics relating to the question. | 1 | 0 | Fail | Says ‘what kind of place are you looking for, tobacconists or playgrounds?’  Clicking on playgrounds and Siri says ‘I couldn’t find any matching playgrounds’ |  | Google > GA=Fail, Siri (fail) |
| Q75. Where is the nearest smokefree park | 1 | Fail | First link is a Google map showing the ‘old breadshop backpackers’ in Reefton, which is very close, but no information on whether it is smokefree, and it is not a park. | 0 | 0 | Fail | First item is information about the nearby Slab Creek camping area. No mention of its smoke-free status. | 1 | 0 | Fail | Says ‘ok heres what I found’ the link is to Haven Road Store and Lotto ‘tobacco shop’ 210km away in Nelson. | All Fail |  |
| Q75 | 0 | NA | Gives the nearest playground which also happens to be in the nearest smokefree park (Karori Park, 230m away) | 0 | 1 | Fail | First link is a new article but which has nothing about parks. http://i.stuff.co.nz/dominion-post/news/wellington/80587424/Wellington-city-council-announces-new-smokefree-action-plan | 1 | 0 | Fail | First link is to an e-cigarette shop in Wellington CBD |  | Google > GA=Fail, Siri (fail) |
| Q76. Where is the nearest smokefree beach | 1 | NA | First link is to a Stuff article titled ‘Caroline Bay is now the South Island’s first smokefree beach’ https://www.stuff.co.nz/timaru-herald/news/94830867/caroline-bay-now-the-south-islands-first-smokefree-beach article dated July 2017 | 0 | 0 | NA | First link is to a Stuff article titled ‘Caroline Bay is now the South Island’s first smokefree beach’ https://www.stuff.co.nz/timaru-herald/news/94830867/caroline-bay-now-the-south-islands-first-smokefree-beach article dated July 2017 | 1 | 0 | NA | Says ‘what kind of place are you looking for?’ and gives option for tobacconists or public beaches’ clicking beaches gets you directions to ‘Little Beach’ 92 km away. No info whether its smokefree. | Google = GA > Siri |  |
| Q76 | 0 | Fails | Goes to an options paper for the Council but with no details on existing local smokefree beaches: https://wellington.govt.nz/~/media/about-wellington/research-and-evaluation/smokefree-report-wellington-otago-uni.pdf | 0 | 1 | Fail | First link is a news article on smokefree options (“Public feedback sought on smokefree options…) but which has nothing about existing smokefree beaches: http://i.stuff.co.nz/national/health/70996937/public-feedback-sought-on-smokefree-options-for-wellington | 1 | 0 | Fail | Finds a beach (Evans Bay) but this is not smokefree |  | All fail |
| Q77. Where is the cheapest place to buy nicotine patches | 1 | C | First link goes to a money saving forum http://forums.moneysavingexpert.com/showthread.php?t=649699 where someone asked the question where to buy cheap patches. One of the comments replying says ‘I got mine free from the doctor’ *Not local information and dated 2007* | 0 | 2 | C | First link goes to a money saving forum http://forums.moneysavingexpert.com/showthread.php?t=649699 where someone asked the question where to buy cheap patches. One of the comments replying says ‘I got mine free from the doctor’ *Not local information and dated 2007* | 1 | 0 | Fail | Says ‘what kind of businesses are you looking for’ | Google = GA > Siri (fail) |  |
| Q77 | 2 | C | First link goes to a money saving forum http://forums.moneysavingexpert.com/showthread.php?t=649699 where someone asked the question where to buy cheap patches. One of the comments replying says ‘I got mine free from the doctor’ *Not local information and dated 2007* | 0 | 3 | C | First link goes to a money saving forum http://forums.moneysavingexpert.com/showthread.php?t=649699 where someone asked the question where to buy cheap patches. One of the comments replying says ‘I got mine free from the doctor’ *Not local information and dated 2007* | 1 | 0 | Fail | Says ‘what kind of businesses are you looking for’ |  | Google = GA > Siri (fail) |
| Q78. Where is the cheapest place to buy e-cigarette refills | 0 | NA | First link is to https://www.electrictobacconist.com/refills-c2 an online store with a range of relevant products and shipping available. | 0 | 0 | NA | First link goes to ‘Vapo Vape NZ’ which sells vaping products online. | 1 | 0 | NA | Says ‘OK Matt, heres what I found’ and gives directions to haven road store and lotto tobacco shop in Nelson 210 km away. | Siri > GA > Google |  |
| Q78 | 0 | NA | An online e-cigarette retailer in NZ: http://www.cosmicnz.co.nz/e-cigarette (does list a Wellington based shop) | 0 | 0 | NA | First link goes to ‘Vapo Vape NZ’ which sells vaping products online. – but no local stores shown. | 1 | 0 | Fail | Says ‘OK Nick, heres what I found’ and gives directions to haven road store and lotto tobacco shop in Nelson 210 km away. |  | Google > GA > Siri (fail) |
| Q79. Find an online calculator for the cost of smoking | 1 | A | First link is the HPA/Smokefree calculator specific for NZ: http://www.smokefree.org.nz/smoking-its-effects/cost-of-smoking/cost-of-smoking-calculator | 0 | 1 | A | First link is the HPA/Smokefree calculator specific for NZ: http://www.smokefree.org.nz/smoking-its-effects/cost-of-smoking/cost-of-smoking-calculator | 1 | 1 | A | Says ‘ok, I found this on the web for…’  First link is the HPA/Smokefree calculator: http://www.smokefree.org.nz/smoking-its-effects/cost-of-smoking/cost-of-smoking-calculator | Google = GA = Siri |  |
| Q79 | 1 | A | First link is the HPA/Smokefree calculator specific for NZ: http://www.smokefree.org.nz/smoking-its-effects/cost-of-smoking/cost-of-smoking-calculator | 0 | 1 | A | First link is the HPA/Smokefree calculator specific for NZ: http://www.smokefree.org.nz/smoking-its-effects/cost-of-smoking/cost-of-smoking-calculator | 1 | 0 | A | Says ‘ok, I found this on the web for…’  First link is the HPA/Smokefree calculator: http://www.smokefree.org.nz/smoking-its-effects/cost-of-smoking/cost-of-smoking-calculator |  | Google = GA = Siri |
| Q80. How do you say ‘where can I buy nicotine patches’ in the French language | 0 | NA | Google information box says: “où puis-je acheter des correctifs de nicotine 'dans le  - which is correct but it includes the words “in the” from the question. | 1 | 0 | NA | Reads the french aloud. Google information box says: “où puis-je acheter des correctifs de nicotine 'dans le  - which is correct but it includes the words “in the” from the question. | 1 | 2 | NA | Says ‘ok I found this on the web for…’  Google information box says: “où puis-je acheter des correctifs de nicotine 'dans le  - which is correct but it includes the words “in the” from the question. | Google = GA = Siri |  |
| Q80 | 0 | NA | Google information box says: “où puis-je acheter des correctifs de nicotine 'dans le  - which is correct but it includes the words “in the” from the question. | 1 | 3 | NA | Reads the french aloud. Google information box says: “où puis-je acheter des correctifs de nicotine 'dans le  - which is correct but it includes the words “in the” from the question. | 2 | 0 | NA | Says “I can’t translate from New Zealand English yet. Sorry about that.” |  | Google = GA > Siri (fail) |
